# Recent Advances in Solvents for the Dissolution, Shaping and Derivatization of Cellulose: Quaternary Ammonium Electrolytes and their Solutions in Water and Molecular Solvents

**DOI:** 10.3390/molecules23030511

**Published:** 2018-02-25

**Authors:** Marc Kostag, Kerstin Jedvert, Christian Achtel, Thomas Heinze, Omar A. El Seoud

**Affiliations:** 1 Institute of Chemistry, University of São Paulo, Av. Prof. Lineu Prestes 748, 05508-000 São Paulo, SP, Brazil; marc_kostag@usp.br; 2 Bio-based Fibres, Swerea IVF, P.O. Box 104, SE-431 22 Mölndal, Sweden; kerstin.jedvert@swerea.se; 3 Centre of Excellence for Polysaccharide Research, Institute of Organic Chemistry and Macromolecular Chemistry, Friedrich Schiller University of Jena, Humboldtstraße 10, 07743 Jena, Germany; christian.achtel@uni-jena.de (C.A.); thomas.heinze@uni-jena.de (T.H.)

**Keywords:** quaternary ammonium electrolytes, molecular solvents, super bases, cellulose dissolution mechanism, cellulose derivatization, cellulose shaping, biomass conversion

## Abstract

There is a sustained interest in developing solvents for physically dissolving cellulose, i.e., without covalent bond formation. The use of ionic liquids, ILs, has generated much interest because of their structural versatility that results in efficiency as cellulose solvents. Despite some limitations, imidazole-based ILs have received most of the scientific community’s attention. The objective of the present review is to show the advantages of using quaternary ammonium electrolytes, QAEs, including salts of super bases, as solvents for cellulose dissolution, shaping, and derivatization, and as a result, increase the interest in further investigation of these important solvents. QAEs share with ILs structural versatility; many are liquids at room temperature or are soluble in water and molecular solvents (MSs), in particular dimethyl sulfoxide. In this review we first give a historical background on the use of QAEs in cellulose chemistry, and then discuss the common, relatively simple strategies for their synthesis. We discuss the mechanism of cellulose dissolution by QAEs, neat or as solutions in MSs and water, with emphasis on the relevance to cellulose dissolution efficiency of the charge and structure of the cation and. We then discuss the use of cellulose solutions in these solvents for its derivatization under homogeneous and heterogeneous conditions. The products of interest are cellulose esters and ethers; our emphasis is on the role of solvent and possible side reactions. The final part is concerned with the use of cellulose dopes in these solvents for its shaping as fibers, a field with potential commercial application.

## 1. Introduction

The demand for cellulosic fibers—natural and man-made—is increasing continually because of world population growth. Cotton production, however will not meet this increased demand. Consequently, a rational strategy to close this “cellulosic fiber gap” is to increase the production of man-made cellulosic fibers, following the principles of green chemistry [1,2,3]. Industrially, fibers, e.g., from wood cellulose, are obtained from Viscose (cellulose xanthate in alkali solution) by extrusion in acid bath to produce the fiber Rayon [4,5,6], or by regeneration of cellulose solutions in *N*-methylmorpholine *N*-oxide (NMMO) hydrate in an aqueous bath to produce the fiber Lyocell [7,8,9,10,11]. The limitations of both solvents and processes, coupled with the increased emphasis on sustainability have prompted the search for alternative greener substitutes. This necessity is the impetus for the continued interest in developing new cellulose solvents, despite the existence of several ones in commercial use [12,13,14,15,16]. Imidazole-based ILs have gained importance in the areas of cellulose dissolution, processing and derivatization, resulting in a large number of publications, including review articles [17,18,19,20,21,22,23,24,25,26,27,28,29,30,31,32,33,34,35,36,37,38,39,40,41,42,43,44,45,46]. Some quaternary ammonium electrolytes (QAEs) have melting points below 100 °C and can, therefore, be classified as a subclass of ionic liquids (ILs). In the present review, we cover QAEs that do not bear heterocyclic rings, e.g., tetraalkylammonium halides. We also discuss deep eutectic solvents (DES), and a special class of heterocyclic derivatives, namely, the salts obtained by neutralization of heterocyclic superbases. After giving a short account on their history, we dwell on recent advances in their use as solvents for cellulose dissolution, derivatization, and shaping.

**Figure 1:**
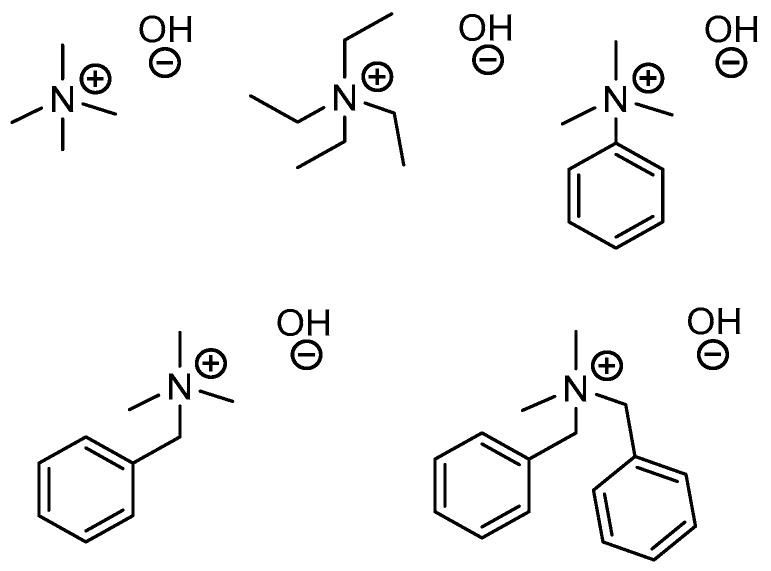
Top: from Lilienfeld’s 1924 patented electrolytes for cellulose dissolution [48,49]; bottom: the first commercially available ammonium hydroxides for cellulose dissolution, known as Triton B (left) and Triton F (right) [51].

The use of aqueous solutions of cellulose in urea, urethane, guanidine and their derivatives was already reported in 1924 [47]. Alkali-containing [48,49] and alkali-free aqueous quaternary ammonium hydroxides were reported as cellulose solvents as well (Figure 1, top) [50]. Benzyl-substituted quaternary ammonium hydroxides were found to be more efficient cellulose solvents [51,52], leading to the discovery of the well-known electrolytes Triton B and Triton F (Figure 1, bottom) [53,54]. Nevertheless, these cellulose solvents did not have technical importance, mainly due to their high water content, and use of expensive silver oxide in their synthesis (for halide/hydroxide anion exchange).

The inorganic compound ammonium thiocyanate in combination with ammonia was also reported as a cellulose solvent quite early [55]. It was shown that several freeze-thawing cycles enhanced cellulose dissolution [56,57]. Because water and ammonia are not suitable for obtaining some cellulose derivatives, and inorganic electrolytes have limited solubility in organic solvents, organic ammonium salts became the logical candidates to focus on. Hence, in 1986 the binary mixture of tetraethylammonium chloride (N_2222_Cl) in DMSO was reported to dissolve up to 12 wt% cellulose at 100 °C [58]. Pyridine and DMF were suitable MSs as well. This solvent system (QAE/MS) is the organic counterpart of the solvent LiCl/DMAc that was introduced a few years earlier [59].

**Figure 2:**
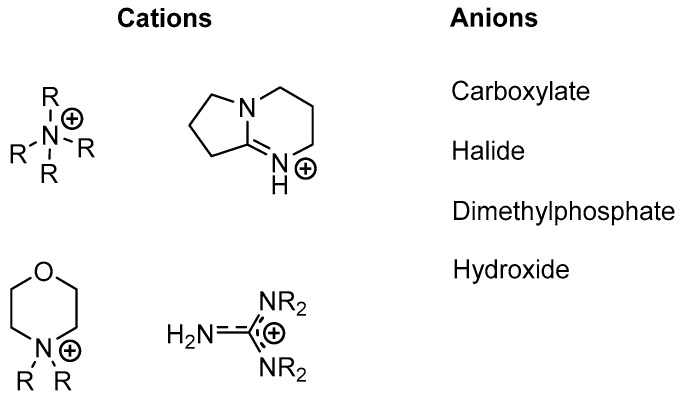
The general molecular structures of the electrolytes reviewed: ammonium, morpholinium, amindinium and guanidinium cations and the most commonly used anions.

Introduction of imidazole-based ILs as cellulose solvents [17], increased noticeably the effort to develop new solvents for cellulose. The structural versatility of ILs is shared by simple QAEs that are used alone, or as solutions in water and MSs. In the present review, we focus on QAE-based cellulose solvents; these have received less attention than ILs. The QAE systems that we address include quaternary ammonium carboxylates, halides, hydroxides, and phosphates. We also include salts of superbases, e.g., tetramethylguanidine (TMG) and 1,5-diazabicyclo[[4.3.0]non-5-ene (DBN) although for these salts the distinction between being ILs or QAEs is rather operational (Figure 2). Where required, we show results of ILs because both classes of compounds share common ground, e.g., anion-basicity dependent cellulose dissolution. We hope that this account will increase the interest in the use of these versatile and efficient cellulose solvent systems.

## 2. General Synthesis Strategies for Quaternary Ammonium Electrolytes (QAEs)

QAEs are easily available via quaternization of amines; many of them are available commercially, or can be obtained by Hofmann degradation of amides [60]. The simplest derivatives are the ammonium salts obtained by neutralizing the amine, usually with a mineral acid. However, attempts to dissolve cellulose in these molten electrolytes (e.g., trimethyl and triethylammonium chloride) were unsuccessful, and led to fast darkening of the cellulose solutions [61]. The association between the ammonium cation and the chloride anion is probably more efficient than the interaction of cellulose hydroxyl groups with the chloride. The presence of solvated protons in the medium leads to acid-catalyzed cellulose degradation, hence solution darkening.

**Scheme 1:**
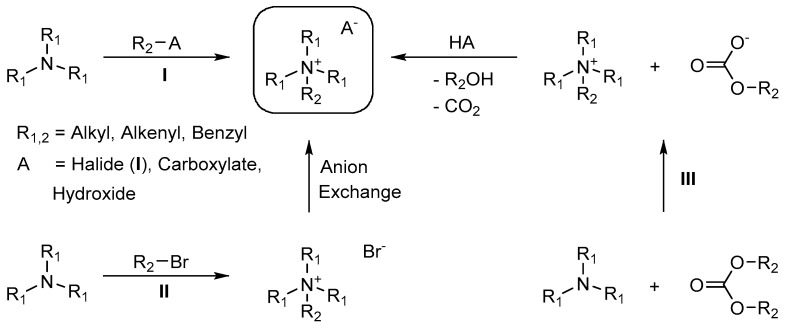
Synthesis of quaternary ammonium electrolytes by quaternization of tertiary amines with organic halides (**I**, **II**) or dialkyl carbonates (**III**).

The deleterious effect on cellulose integrity of the solvated protons is eliminated by quaternization of amines. Many *N,N,N*-dimethylalkylamines are commercially available or can be easily prepared, e.g., via exhaustive alkylation at the nitrogen and reduction of the intermediate imine (Eschweiler-Clarke reaction) [62,63,64]. The subsequent quaternization can be carried out by the Menshutkin reaction (Scheme 1, path **I**, if R_2_ = alkyl, but in principle R_1_ and R_2_ can be any alkyl, alkenyl or benzyl group) [65,66]. In this reaction, the molecular structures of the alkyl halides and amines influence the reaction yield as well as its mechanism. For example, for S_N_2 reactions in aprotic solvents the order of reactivity of the amines is: primary > secondary > tertiary; whereas that of the halides is RI > RBr > RCl. Energetic conditions (high temperature and/or pressure) are required for the reaction of sterically hindered amines (e.g., tribenzylamine) with alkyl halides; tertiary halides react with amines essentially by the S_N_1 mechanism [67]. The Menshutkin reaction produces the so-called first generation QAEs. Second generation QAEs are very important because of the relevance of basicity of the anion to cellulose dissolution [68,69]. Exchange of the halide anions with, e.g., carboxylates is carried out with anion exchange resins (usually macroporous), *via* the sequence shown in path **II** of Scheme 1 (halide → hydroxide → carboxylate). We found that direct anion exchange (halide → carboxylate) is complete, e.g., for chloride → acetate [70]. For efficient methylation or ethylation of amines the use of alkyl carbonates is possible (Scheme 1, path **III**) [71]; the resulting carbonate is converted into other anions by reaction with the appropriate acid. Alkyl phosphates, and alkyl sulfates are also used for amine alkylation at the nitrogen [72].

Because neat (molten) QAEs are also used to dissolve cellulose [61,73] a comment on the effect of the anion and cation on their melting points is in order. As shown for ILs, quaternary ammonium halides are liquids at room temperature if the Gibbs free energy of fusion is negative, resulting in the liquid state being thermodynamically favored [74]. As a rule of thumb, the melting points decrease with increasing cation size. In addition, asymmetric substitution or branched alkyl chains lead to lower melting points. It is suggested that one or two longer chains, e.g. *n*-octyl, with two or three shorter chains, e.g. *n-*butyl should be beneficial for lowering the melting point of the QAE [75]. Considering the same cation with halide anions, the melting points decrease in the order Cl > Br > I [76]; the melting points of the carboxylates are usually lower than the corresponding halides. Apparently, systematic variation of melting points along a homologous series of electrolytes does not always work. They can show scatter depending on the combination of cations and anions [77]. 

## 3. Mechanism of Cellulose Dissolution by Neat QAEs, Their Solutions in Water and Molecular Solvents

### 3.1. Solvents for Dissolution, Regeneration and Derivatization of Cellulose

It is known that the (physical) dissolution of cellulose in any solvent relies on the disruption of the inter- and intramolecular hydrogen bonding between the hydroxyl groups of the anhydroglucose unit (AGU), as well as the solvophobic interactions present [78,79,80]. This is essentially achieved by a combination of cooperative action of the electrolyte solvated ion pair on bonding in cellulose, sufficient basicity of the anion and adequate volume of the electrolyte/solvent complex [81]. Spectroscopic techniques, in particular NMR [82,83,84,85] and IR spectroscopy [85,86], rheology [87,88] and theoretical calculations [83,89,90] shed light on the mechanism of cellulose dissolution and the relationship between the solvent efficiency, the nature of the MS and ions of the QAE. We address these aspects below by examining separately the effects of anion and cation.

#### 3.1.1. Basicity and volume of the anion

Regarding anions, disruption of hydrogen bonds in cellulose increases as a function of increasing the charge density (or hardness), and decreasing the volume of anion. As a result, the biopolymer dissolution capacity of QAE solutions is expected to be related to the Hofmeister series [91]. The interactions of 2,2-bis(hydroxymethyl)-1,3-propanediol (a model for cellulose) with a series of tetra-(*n*-butyl)ammonium salts (TBAX)/DMSO (X = anion) was studied using IR spectroscopy [85]. Note that hydrogen bonding between TBAX and the hydroxyl group of the polyol (TBAX···HO-polyol) results in a red shift of the frequency of the O-H stretching vibration (ῦ(ν_O-H_)) whose magnitude increases as a function of increasing the hydrogen bond strength. Relative to ῦ(ν_O-H_) of the parent polyol in DMSO, the only peak that showed red shift is that in presence of tetra-(*n*-butyl)ammonium fluoride (TBAF). For the TBAX series the values of ῦ(ν_O-H_) were in the order X = F^−^ < Cl^−^ < Br^−^ < I^−^ . That is, the interactions X^−^···HO-polyol increased as a function of *increasing* the charge density and *decreasing* the volume of the halide ion. A similar trend was observed for the same polyol in solutions of the ILs 1-ethyl-3-methylimidazolium X (EtMeImX, X = halide) in DMSO [89]. The values of Δῦ(ν_O-H_) (= ῦ(ν_O-H_) in DMSO - ῦ(ν_O-H_) in QAE/DMSO) obeyed the Hofmeister series, as shown by the following order: F^−^ > CH_3_CO_2_^−^ > (C_2_H_5_)_2_PO_4_^−^ > Cl^−^ > SCN^−^ > Br^−^ > I^−^ > C_2_H_5_SO_4_^−^. These results bear on cellulose dissolution in QAEs/DMSO; the biopolymer is easily soluble in TBAF·3H_2_O/DMSO but not in other TBAX/DMSO with less basic anions (X = Cl^−^, Br^−^, I^−^) [86].

The extensive use of TBAF·3H_2_O/DMSO as solvent for cellulose dissolution and derivatization merits additional comments. At room temperature, anhydrous TBAF is the only R_4_NX that is liquid; it shares with ILs the property of negative energy of fusion [74]. However, obtaining anhydrous TBAF by dehydration of TBAF·3H_2_O without extensive Hofmann degradation is laborious [86]; its preparation in situ from the reaction between tetra-(*n*-butyl)ammonium cyanide (TBACN) and C_6_F_6_ is expensive, [92] and the resulting TBAF/DMSO is relatively unstable [93]. Consequently, the use of commercially available, solid (m.p. 58–60 °C) TBAF·3H_2_O/DMSO is convenient. At first glance, the efficiency of TBAF·3H_2_O/DMSO as solvent for cellulose may seem surprising because the free energies of transfer of the (F^−^) from water to virtually all dipolar aprotic solvents are positive, i.e., unfavorable due to the strong solvation of this anion by hydrogen bonding to water [94]. However TBAF·3H_2_O is still a reasonably powerful nucleophile and base, although both properties are dramatically attenuated as a function of increasing the water content of this QAE [95,96]. The potential problem with the use of this electrolyte, however, is the effect of its (uncontrolled) water content on the results, e.g., the reproducibility of the degree of substitution (DS) when different electrolyte batches are employed, or when the same electrolyte sample is employed over a relatively long period. In contact with air, solution of TBAF·3H_2_O/DMSO absorbs water continuously for hours [97]. Additionally, the water of hydration present in this QAE leads to hydrolysis of the acylating agent. This undesirable side reaction is general-base catalyzed by (F^−^). The deleterious effect of this water absorption was neatly shown by measuring the dependence of the chemical shifts and bandwidths of the water protons and (F^−^) as a function of the water content in cellulose TBAF/DMSO solutions. The results suggest the formation of strong Cel-OH···F^−^ bonds, leading to the breakdown of the cellulose-cellulose hydrogen bonds, hence biopolymer dissolution. The latter is favored by electrostatic repulsion between the negatively charged Cel-OH···F^−^ chains [82]. Each negative chain is most certainly surrounded by a sheath of the TBA^+^ cation. The synergism between *electrostatic repulsion* of Cel-OH···anion, and *steric repulsion* of Cel-OH···anion/cation complexes appear to prevent association between cellulose chains and favor a molecularly dissolved state as shown for QAEs [83], and, e.g., for the ILs BuMeIm [26,84,98,99,100,101,102,103] or EtMeIm cation [104,105,106,107,108].

**Figure 3:**
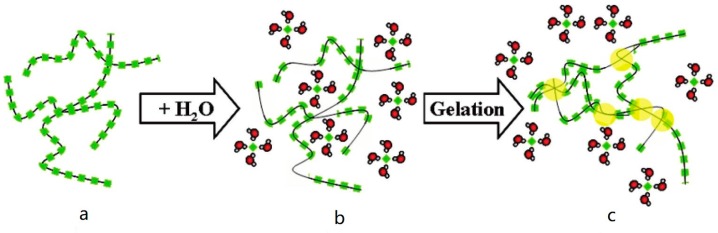
NMR-based illustration of the mechanism of water-induced gelation of cellulose dissolved in TBAF/DMSO. The symbols are: red dots = water molecules, green squares = F^−^ (associated with cellulose in (**a**), solvated by water in (**b**), partially exposed cellulose chains, which leads to reaggregation (**c**), black lines = cellulose chains; yellow circles = hydrogen bond regions between cellulose chains; reprinted with permission from [82], Copyright (2009) American Chemical Society.

Small concentrations of water strongly solvate the fluoride ions, inhibiting them from association with the cellulose chains. This allows the reformation of the cellulose-cellulose hydrogen bonds, which leads to highly viscous solutions or gel formation. These changes in the physical state of the solution are schematically depicted in Figure 3. Part (a) of Figure 3 shows the cellulose in dry TBAF/DMSO solution; part (b) shows preferential solvation of (F^−^) by the added water and its removal from cellulose. This lead, finally, to solution gelation due to reestablishing strong hydrogen bonding and hydrophobic interactions between the cellulose chains (c) [82]. Quaternary ammonium fluorides of much less water content were prepared by a simple protocol, e.g., N_Al4_F·H_2_O/DMSO and N_11Bz2_F·0.1 H_2_O/DMSO (Al and Bz refer to allyl and benzyl group, respectively). Both QAE·xH_2_O/ DMSO dissolve cellulose and the biopolymer was efficiently acylated in these solvents (see Section 4.3 *Ammonium fluorides*) [109,110]. Interestingly, tolerance for water as a non-solvent for cellulose dissolution in tetra-(*n*-butyl)ammonium acetate (TBAAcO)/DMSO is twice as high as tolerance for ethanol (calculated on a molar basis). Based on theoretical calculations, it was suggested that the higher tolerance to water is due to its more efficient hydrogen bond interactions that improve solvation of cellulose and, thereby, marginally favor dissolution [111]. The higher tolerance toward water may be traced to the fact that water-DMSO interactions are stronger than water-water interactions [112,113]. The influence of water and other non-solvents in imidazole-based IL solutions on the cellulose solubility is reported as well, e.g. in [102,114] but is outside the scope of the present review.

In summary, the hygroscopic nature of solutions of these QAEs/MS should never be underestimated because uncontrolled water contents have deleterious effects on their efficiency as cellulose solvents, and the economy of the process due to hydrolysis of the acylating agent. Additionally, effort should be made to control the purity, including water contents of these solutions as stressed repeatedly for ILs and QAEs [17,115,116,117,118]. In case of QAEs, the presence of small concentrations of KBr in the commercial aqueous tetra-(*n*-butyl)ammonium hydroxide (TBAOH) and TBAAcO/LiCl/DMSO decreased the solubility of cellulose, presumably due to the formation of (K**^+^**)-mediated complexes between cellulose chains. Addition of (K**^+^**)-complexing crown ether 18-crown-6 resulted in clear biopolymer solutions in both cases and increased the concentration of dissolved cellulose, although this represents an expensive solution to the QAE purity problem (see also Section 4.2 *Ammonium carboxylates*) [119,120].

The carboxylate group is also a hard base. Therefore, QAEs bearing the carboxylate moiety were successfully tested as solvents for cellulose, with emphasis on TBAAcO/DMSO. Indeed, cellulose fibers from this solvent were obtained without noticeable degradation or (slow) formation of cellulose acetate during dissolution (see Section 4.2 *Ammonium carboxylates*) [121]. The latter (acetylation) side reaction occurs during acylation of cellulose in pure EtMeImAcO [122].

**Figure 4:**
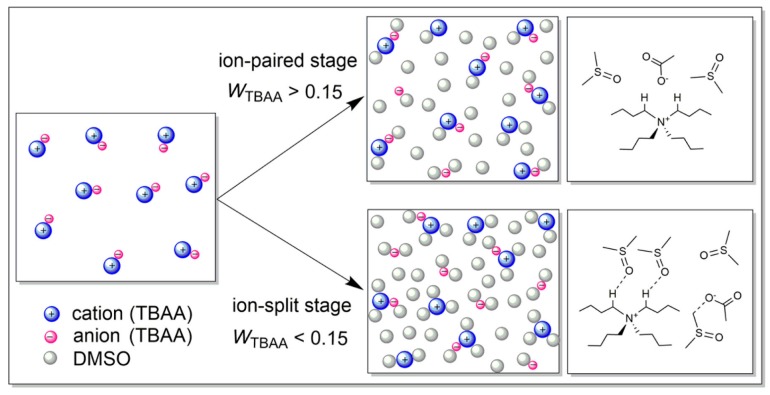
Schematic representation of the association of cellulose with the ions of TBAAcO as a function of the mass ratio W_TBAAcO_ of the QAE with DMSO; reprinted with permission from [85], Copyright (2016) American Chemical Society.

Different types of cellulose (MCC, cotton, regenerated cellulose) dissolve in TBAAcO/DMSO at 25 °C [85]. The dependence of wt% dissolved cellulose and dissolution time on the electrolyte mass ratio W_TBAAcO_ [mass of TBAAcO/(mass of TBAAcO + mass of DMSO)] was studied. All results showed increases in cellulose dissolution until W_TBAAcO_ = 0.15, then a decrease at higher W_TBAAcO_. The dependence of solvent efficiency on QAE is schematically represented in Figure 4, where the degree of dissociation of the QAE is larger at W_TBAAcO_ < 0.15 than at W_TBAAcO_ > 0.15 because of the association of QAE ions with cellulose. The authors corroborated this conclusion by the following pieces of evidence: (i) Dependence of solution conductivity (cellulose/TBAAcO/DMSO) on W_TBAAcO_ that showed a rapid increase until W_TBAAcO_ ~ 0.15 followed by subsequent slower increase; (ii) dependence of ^1^H and ^13^C-NMR chemical shifts of the cation and anion on W_TBAAcO_ in DMSO-d_6_, with a “inflection point” at W_TBAAcO_ ~ 0.15; (iii) dependence of the ^1^H- and ^13^C-NMR chemical shifts of the cation and anion of TBAAcO on [cellobiose], showing large changes until W_TBAAcO_ ~ 0.15, followed by small changes at W_TBAAcO_ > 0.15; (iv) dependence of ῦ(ν_S=O_) of DMSO on [cellobiose] (decrease in ῦ(ν_S=O_) as a function of increasing [cellobiose]. Thus, the balance between ion concentration and mobility is crucial to cellulose dissolution. This balance is largely controlled by hydrogen bonding and solvophobic interactions. The favorable effect of DMSO is due to viscosity reduction and stabilization of the cellulose-QAE complex [85].

Similar dependence of viscosity and conductivity of cellulose/IL/MS mixtures and the wt% of maximum dissolved cellulose on IL concentration were also observed for AlMeImCl in DMSO [87]. The rheological properties of cellulose/IL/MS mixtures (mostly DMSO as MS) were studied extensively and data regarding viscosity of these solutions are readily available in the literature [123,124,125,126,127,128,129,130,131,132,133,134,135]. The comparison and evaluation of the results is not straightforward because there is no common experimental “protocol” for cellulose dissolution (cellulose type, temperature, IL/MS ratio). The effect of MS on the viscosity of cellulose/IL solution have been reviewed as well [136]. It is noteworthy that the viscosity of QAE/MS solution is important but not a controlling factor because at comparable concentration, the same IL is more efficient in DMSO than in (less viscous) DMAc [88].

Dissolution of cellulose in TBAAcO/DMSO was determined at 60 °C by microscopy and turbidity measurements. The results for three QAEs suggested maximum solubilization at molar ratio QAE/AGU of unity. This conclusion was further corroborated from NMR data. This included ^1^H-NMR chemical shifts of the anion and cation of QAE; diffusion coefficients of the ions of QAE and the MS as a function of the molar ratio QAE/AGU, and the results of molecular dynamics (MD) simulations of the system. The latter calculations indicated binding of the acetate ion to more than one hydroxyl group of the AGU. The calculated contact time between the acetate ion and cellulose is at least an order of magnitude longer than the contact time between any other pair of species in the system (cation-cellulose, cation-DMSO, DMSO-DMSO), a clear indication of the strength of anion···HO-Cel hydrogen bonding [83]. The simultaneous binding of the anion to more than one hydroxyl group in the same AGU was also advanced to explain the efficiency as cellulose solvents of R_4_NF·xH**_2_**O/DMSO and AlMeImCl/DMSO. For both solvents, the halide ion binds simultaneously to C2-OH and C3-OH of the AGU [88,110].

We now address the effects of the volume and charge density of the cation because these variables affect the association anion-cation of the QAE and its (solvophobic) interactions with cellulose, thus bear on its efficiency of as solvent.

#### 3.1.2. Acidity and Volume of the Cation

**Figure 5:**
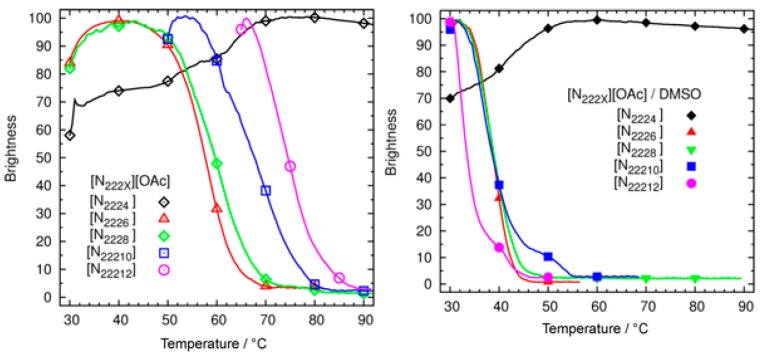
Comparison of the temperature of dissolution of 10 wt % microcrystalline cellulose in pure N_222R_AcO (top) and in mixtures of N_222R_AcO/DMSO (80/20 wt%, bottom); reprinted (adapted) with permission from [73], Copyright (2017) Wiley-VCH Verlag.

The quaternary ammonium ions are soft acids [137]. Their volumes are important in determining anion-cation interactions whose strength are determinant to cellulose dissolution, as also shown for IL cations [101,138,139]. This was shown by the fact that TBAF·3H**_2_**O/DMSO dissolves cellulose easily, whereas the biopolymer is insoluble in N_1111_F/DMSO and only marginally soluble in N_111Bz_F/DMSO. This was explained on the bases of solubility of these QAEs in DMSO at room temperature: 0.94, 0.025 mol/L and negligible, respectively [93]. As shown above, electrolyte solubility and dissociation into free ions in the MS are required for efficient ion/AGU interactions, leading to cellulose dissolution. This idea was corroborated by (DFT) theoretical calculations. Due to the weak interaction of (F^−^) with the voluminous (TBA^+^) cation of TBAF, the anion transfers significant amount of charge into the antibonding orbital of the Cel-OH groups leading to the disruption of the hydrogen bonding and cellulose dissolution. Due to the smaller volume of (N_1111_^+^) of N_1111_F, the (F**^−^**) transfers more negative charge into the positively charged cation, hence cannot disrupt the hydrogen bonding of cellulose; the biopolymer is insoluble [89]. In a systematic study on the effect of QAE cation volume on cellulose dissolution, neat N_222R_AcO, N_222R_PrO, N_333R_AcO, N_333R_PrO and their solutions in DMSO were studied (N_222R_ and N_333R_ refer to derivatives of triethylamine and tri-(*n*-propyl)amine, respectively; R = *n*-alkyl, from butyl to dodecyl). Neat QAE with (R = *n*-butyl) did not dissolve cellulose, independent of the structure of the parent tertiary amine (N_222_ or N_333_). In general, addition of DMSO increased cellulose solubility. The efficient QAEs are those with R longer than *n*-hexyl, as illustrated in Figure 5. For the most efficient QAE N_2228_AcO at 80 °C the calculated molar ratio QAE/AGU is 4.8 (neat QAE) and 2.1 (20 wt% DMSO) [73]. The latter (electrolyte/AGU) ratio is smaller than that observed for TBAAcO/DMSO [83], and may reflect the effect of the cation steric hindrance in both QAEs.

**Figure 6:**
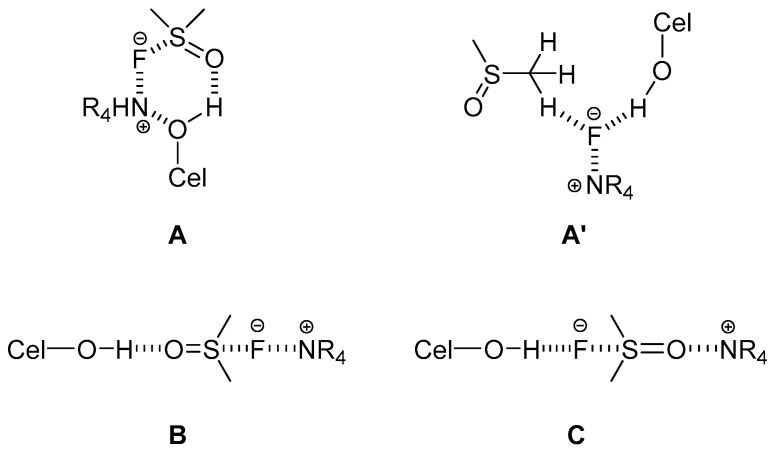
Schematic representations of the structures of interest, including, the starting geometries of the aggregates (**A**–**C**), and (**A**’) an optimized geometry of **A** [140,141]; redrawn from [90].

Regarding the molecular structures formed in solutions of cellulose/QAE/DMSO quantum chemistry calculations were performed on cellobiose, methylated at the positions *O*-4 and *O*-1 as model for cellulose, and QAEs (N_1111_F, N_Al4_F, N_11Bz2_F, and TBAF)/DMSO. We present the corresponding structures in Figure 6 (see reference [90] for details of these calculations).

In Figure 6, (A) is cyclic whose structure is similar to that indicated elsewhere [140], whereas (B) and (C) are linear with the (F^−^) bridging the sulfur atom of DMSO either to the quaternary nitrogen of the electrolyte, or to one of the OH groups of the cellulose model. Structure (A´) is the theoretically optimized version of (A). Interestingly, during geometry optimization of (A), the initial cyclic aggregates changed to an F^−^-centered structure, with change in connectivity, as shown in structure (A´). Based on the results of these calculations, we suggested that the aggregates formed in the system cellulose model/R_4_NF/DMSO are best represented by (C). This structure is corroborated by the above-mentioned NMR and FTIR data [82,89].

### 3.2. Salts of Super Bases

**Figure 7:**
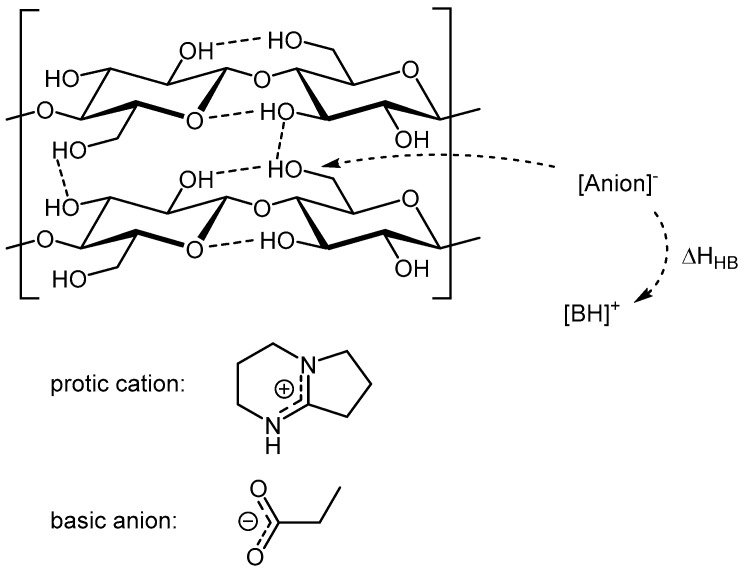
Model for the dissolution of cellulose in protic QAEs on the basis of super bases; redrawn from [143].

A new class of QAEs based on salts of super bases was recently introduced as potential solvents for cellulose [36,142,143,144,145,146]. These QAEs are prepared by neutralizing superbases, e.g., 1,1,3,3-tetramethylguanidine (TMG), 1,5-diazabicyclo-[[4.3.0]non-5-ene (DBN), 1,8-diazabicyclo- [[5.4.0]undec-7-ene (DBU) with carboxylic acids, such as acetic or propionic acid. The mechanism of cellulose dissolution is mainly connected to the basic moieties of the electrolytes, which can disrupt the strong intra- and intermolecular interactions within the cellulose structure, as shown in Figure 7. The role of solvent descriptors for regeneration of cellulose from EtMeImAcO, TMG PrO and TMG AcO was also compared with other cellulose solvents, e.g., NMMO and LiCl/DMAc [147]. It was found that solvent basicity (*SB*) was the property that changed most (almost a linear decrease) upon addition of water. However, the ability of the mixtures to dissolve cellulose was best explained by the net basicity, i.e. (*SB-SA*), rather than *SB* alone. A window for regeneration of cellulose was suggested (SB < 0.8 and SB-SA < 0.35). Cellulose regeneration was divided into four stages: gelation, particle formation, biopolymer regeneration with adsorbed IL, removal of the latter by washing. It was also shown that there TMG-based solvents were more sensitive towards water during cellulose dissolution than its regeneration. [86] 

### 3.3. Aqueous Solvents for Cellulose Dissolution and Regeneration

Because the above-mentioned QAEs solutions are alkali-free, they are suitable for cellulose dissolution, regeneration and derivatization with reagents that are subject to hydrolysis, e.g., carboxylic acid anhydrides and acyl chlorides. We comment now on another class of efficient cellulose solvents, quaternary ammonium- and phosphonium hydroxides because their larger capacity of cellulose dissolution at room temperature enhances their potential use in cellulose regeneration. Additionally, dissolution by these systems is clearly linked to the amphiphilic characters of cellulose and the QAE. Unlike ILs, the presence of water in the solvent is not a barrier to cellulose dissolution. Thus tetra-(*n*-butyl)phosphonium hydroxide TBPOH containing 40 wt% water dissolves 20 wt% cellulose within 5 min at 25 °C under stirring. The interaction of the hydroxide anions with the hydroxyl groups of the AGU of MCC was demonstrated by following the chemical shift and line width of the (OH^-^/H_2_O) ^1^H-NMR peak as a function of increasing MCC concentration, and the crystallinity of regenerated cellulose (type I → amorphous) [148]. Aqueous solutions of Bu_4_POH also dissolve wood discs (cedar, pine, polar). The dissolution efficiency depends on the water content of the biomass solution; the latter content can be decreased by a slow “auto-recovery or “self-dehydration” step, namely by storing the solution for 2–10 days at room temperature under controlled humidity [149]. 

The effect of water and cation size on the cellulose dissolution capacity of aqueous R_4_POH at 25 °C were studied in detail by using aqueous solutions of the following QAEs: P_RRRR_ OH (R = ethyl, *n*-butyl, *n*-hexyl) and N_RRRR_OH (R = methyl, ethyl, *n*-butyl, *n*-hexyl). The QAEs with the smallest volumes (e.g., N_1111_OH and N_2222_OH) did not dissolve 0.5 wt% cellulose. The biopolymer dissolution capacity and dissolution time (2–20 min) depended on (*n*), the number of water molecules/cation (fast dissolution at *n* ≤ 15). As discussed above, cellulose dissolution by these alkaline solutions involves proton removal, e.g., from C6-OH of the AGU, leading to chemical shift change of the attached (primary) carbon atom. The values of δ ^13^C-NMR signal of this primary carbon of 10 wt% cellobiose decreased as a function of increasing (*n*); the limit of cellulose dissolution was reached at δ ^13^C of 64.8 ppm (*n* ≈ 15). Below this “threshold” chemical shift, cellobiose is insoluble [150]. 

**Figure 8:**
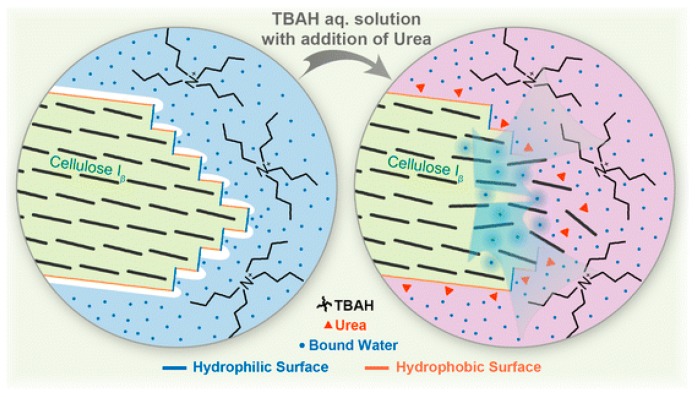
Schematic representation of the mechanism for dissolution of cellulose in TBAOH/urea aqueous solution; reprinted with permission from [154], Copyright (2013) Springer Nature.

The results of several publications highlight the importance of the amphiphilic character of cellulose, and the solvophobic interactions in these QAE solvents. The most relevant result is the effect of urea on dissolution of cellulose. The simplest example is where urea-inorganic base is the solvent. Results of DSC, ^1^H and ^13^C-NMR chemical shifts showed that urea hydrate plays its positive role in dissolution through van der Waals interactions, by accumulating on the hydrophobic face of the AGU to prevent dissolved cellulose chains from re-aggregation [151,152]. The amphiphilic character of both components of the system - cellulose and the QAE - was nicely exploited in using aqueous TBAOH to dissolve cellulose. As shown below, the idea is to “match” cellulose and QAE solution, akin to matching the HLB (hydrophilic-lipophilic balance) of the aqueous phase to that of the oil phase in order to obtain stable oil/water emulsions [153]. The cellulose dissolving power of the aqueous solvent is dependent on QAE concentration and the corresponding molar ratio water/QAE (*n*_W/QAE_) which was divided (based on DSC measurements) into bound and free water, (*n*_bound-W/QAE_) and (*n*_free-W/QAE_), respectively. An increase in QAE concentration decreases the amount of (*n*_bound-W/QAE_), leading to contact between the partially desolvated TBA^+^ cations with concomitant increase in the solvophobic QAE-cellulose interactions. At 60 wt% TBAOH, the value of (*n*_bound-W/QAE_) is ca. 2 and cellulose is easily dissolved by solvophobic interactions. In other words, cellulose dissolution can be induced by “tuning” the hydrophilic-lipophilic character of the QAE solution. Another interesting approach to control this character at a fixed QAE concentration is to use urea or thiourea as additive. Addition of urea to 55 wt% of TBAOH led to an upfield shift of the ^13^C-NMR peaks of (NH_2_)_2_*C*=O and (C_3_H_7_*C*H_2_)_4_NOH, indicating the association urea-QAE with displacement of some of the (*n*_bound-W/QAE_) of the QAE. A similar rational can be applied to the hydrophobic region of the AGU, as depicted in Figure 8 [154].

This amphiphilic aspect was exploited in synthesis where the reagent is not very sensitive to hydrolysis. Thus, aqueous Bu_4_POH was used as solvent for the benzylation of cellulose (reaction with benzyl bromide, 10 min, 20–25 °C, DS_Bz_ ≈ 2.5). The surface activity of Bu_4_POH leads to association with benzyl bromide and with (more hydrophobic) partially benzylated cellulose, leading to products with relatively high DS_Bz_. The above mentioned association (RX-QAE-cellulose ether) results in less hydrolysis of the halide and efficient etherification reaction [155].

## 4. Applications of QAEs for Cellulose Dissolution, Shaping and Derivatization

In principle, the quaternary nitrogen atom of QAEs can be attached to four different substituents (e.g. alkyl, alkenyl, benzyl), leading to structural flexibility; hence their properties can be “tuned” conveniently. They are stable under common conditions for cellulose processing and derivatization, while are not subjected easily to side elimination reactions, unlike 1,3-substituted imidazole-based ILs with their relatively acidic C2-***H*** [156,157,158,159,160,161]. QAEs with various anions were studied, mainly for cellulose dissolution and shaping, and biomass extraction. Cellulose derivatization is reported as well to a lesser extent. In order to give an overview about the QAEs we classified them by their anions. 

### 4.1. Quaternary Ammonium Hydroxides

Parallel to the development of DMSO/TBAF·3H**_2_**O Tanczos et al. studied commercially available, relatively inexpensive tetraalkylammonium hydroxides as activating agents and solvents for cellulose [162]. The ability to swell or dissolve cellulose better than other bases such as NaOH was attributed to penetration of R_4_NOH into cellulose, combined with the amphiphilic character and size of the ammonium cation. The good gelation properties of R_4_NOH were used to obtain fibrous TEMPO oxidized cellulose nano-dispersions in water [163].

An aqueous solution of commercial TBAOH (40 wt%) was found to dissolve 10 wt % cellulose within 24 h at room temperature [119]. As discussed above in Section 3.1.1, 18-crown-6 was added to eliminate the undesirable effect of KBr (a side product in commercial base solutions). Without addition of crown ether, aqueous TBAOH solutions were used to extract cellulose from wheat straw at 60 °C within less than 1 h [164], and dissolve 10 wt % MCC within 2 min at room temperature ([TBAOH] = 50 and 55 wt%, respectively). Analysis of the regenerated polymer confirmed that no degradation or chemical modification occurred. The same group found that TMAOH solutions dissolve 25 wt % MCC in 2 min [165], and extract cellulose from sugarcane bagasse. The latter was achieved by adding 17 to 29 wt % urea to a 40 wt % aqueous solution of TBAOH [154].

An alternative approach was employed to dissolve cellulose using lower concentrations or R_4_NOH [166]. Dissolving grade pulp, 6 wt % was dissolved in a solution containing 70 wt % DMSO, 12 wt % TBAOH, and 18 wt % water. The DMSO concentration could be increased to 90 wt % without cellulose precipitation. After complete solubilization of cellulose, β-cyclodextrin was added to the solution to obtain elastic but robust gels. By varying the cellulose and β-cyclodextrin contents the porosity and mean pore size of the cellulose network was adjusted. These cellulose-based hydrogels could potentially find applications in areas such as removal of heavy metal ions from water, or in controlled drug delivery systems [167]. Examples of the solubility of cellulose in the solvent mixtures mentioned in this section are listed in 

Table 1, where the dissolved cellulose is given in wt%. Because of the differences in the molar masses of the components (AGU, QAE, MS) the use of the mole fraction scale is, to our view, preferable to wt % because it is unambiguous. 

### 4.2. Quaternary Ammonium Carboxylates

QAE carboxylates are, by far, the most studied compounds regarding cellulose dissolution, derivatization and shaping. They have the advantage over hydroxides, that they can be used in non-aqueous solvents. This is an essential prerequisite to perform some cellulose derivatization, e.g., into carboxylic esters. They are also considered to be “*greener*” solvents compared to their corresponding (corrosive) halides (Cl^−^, F^−^).

Molten tetraalkylammonium carboxylates were studied regarding their ability to dissolve and chemically modify cellulose [168]. They were obtained by quaternization of triethylamine or tributylamine with dimethylcarbonate and subsequent conversion with the corresponding carboxylic acid (Scheme 1). Triethylmethyl- and tributylmethylammonium formate (N_1222_Fo, N_1444_Fo) dissolve MCC. A mixture of 8 wt % dilute formic acid (25 wt % aqueous solution) in N_1222_Fo dissolved 10 wt % MCC. The ^13^C-NMR spectrum of this solution revealed the formation of cellulose formate intermadiate during MCC dissolution. As expected, this ester is not stable because pure cellulose was obtained after regeneration of the solution in water. Etherification was performed in these solutions as well. Carboxymethyl cellulose (CMC) with a DS value of 1.55 and a block like distribution of substituents along the polymer backbone was isolated. Hydroxypropylation of MCC, cotton, and spruce sulfite pulp in N_1222_Fo yielded products with a rather low DS (0.22–1.27) in spite of the large excess of molar ratio propylene oxide/cellulose employed (40/1) [169]. 

The biorefinery concept is an expanding research field and a driving force in the development of new solvents for renewable resources, especially cellulose. Thus, QAEs are studied to selectively extract cellulosic material or lignin from biomass. The enzyme catalyzed transformation of cellulose dissolved in IL, e.g., transesterification or hydrolysis, was carried out in bis- and tris(2-methoxyethyl)triethylammonium acetates (N_222 Me(OEt)2_AcO, N_222 Me(OEt)3_AcO) [170]. Many ILs inhibit these reactions because the employed enzymes are readily denatured. Here, the cation structure combined with the basic acetate anion enhances cellulose dissolution (up to 10 wt % MCC) while the low anion concentration stabilizes the enzyme.

A screening of various cations with carboxylate anions as cellulose solvents was conducted by Zhao et al. [118]. Of the QAEs tested only diethyldimethylammonium acetate (N_1122_AcO) dissolved 2 wt % cellulose, the formates, propionates and butyrates did not dissolve cellulose. On the other hand, dialkoxydimethylammonium acetates dissolved up to 18 wt % cellulose (MCC) at 80 °C [171], which underlines the discussion in item 3 above, namely that size, structure, and/or molecular weight of the cation influences the solubilization process to a considerable extent. Additionally, the viscosity of the solutions is significantly lower compared to other IL cellulose solutions, due to the higher flexibility of the alkoxy side-chains.

TBAAcO dissolved in various MS (28/72 by weight), such as DMSO, DMAc, DMF, pyridine, or NMP was reported to dissolve up to 9 wt % MCC within a few minutes at 60 °C [172]. In TBAAcO/DMSO the dissolution occurred faster than in the other mixtures. The cellulose containing solutions were also used to produce fibers and membranes. Cellulose acetate and butyrate with DS values of around 3 and 2, respectively, were obtained by acylation in this solvent.

**Figure 9:**
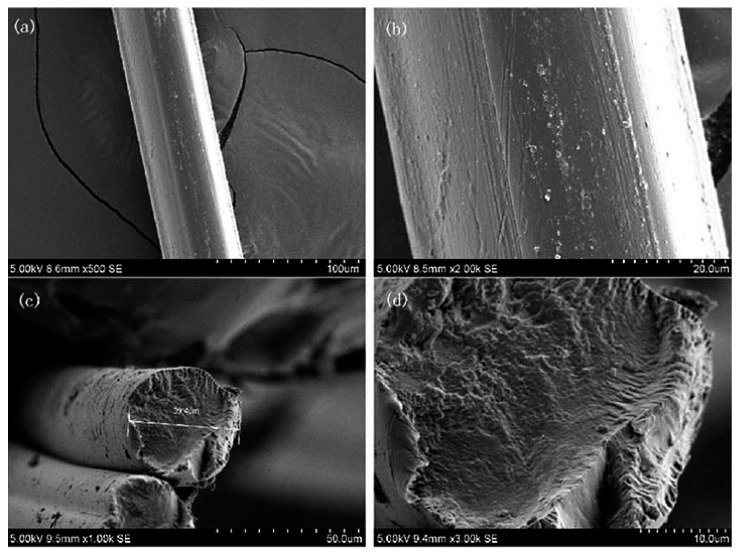
Scanning electron microscope images of cellulose fibers prepared from TBAAcO/DMSO/18-crown-6: (**a**) side surface section, (**b**) magnified side surface section, (**c**) cross-section with diameter, (**d**) magnified cross-section; reproduced from [120] with permission of The Royal Society of Chemistry.

Miao et al. used TBAAcO and mixtures thereof as cellulose solvents as well [120]. Based on the results of DMSO/TBAF·3H_2_O [173] and TBAOH [119] the introduction of the acetate anion was expected to yield a promising cellulose solvent. However, neat TBAAcO did not dissolve cellulose, addition of DMSO was required [120]. The dissolution optimum for cellulose was found when the binary mixture of DMSO contained 15–20 wt % TBAAcO, vide Figure 4 [85]. Here cellulose (8 wt%) was dissolved at room temperature within 2 min. Increasing the temperature to 40 °C resulted in a noticeable increase of cellulose solvent efficiency. Thus 11, 22, 33 wt % TBAAcO in DMSO dissolved 6, 22, and 33 wt % MCC. These QAE concentrations correspond to 1 mol acetate anion/AGU due to hydrogen bonding of the acetate to more than one hydroxyl group of the AGU, vide 3.1.1. [83]. At 40 °C, up to 20 wt % MCC dissolved in the TBAAcO/DMSO/18-crown-6 mixture (2/7/1 by weight). Additionally, high molecular weight celluloses (DP = 830) and lignin were also soluble in the same mixture. The clear and viscous solutions of cellulose in TBAAcO/DMSO/18-crown-6 proved to be suitable for wet-spinning and the cellulose fibers were regenerated in ethanol at room temperature. The fibers showed more amorphous than crystalline cellulose structure based on MAS-NMR and XRD analyses, and they were homogeneous with smooth surfaces and diameters around 40 μm (Figure 9) [120]. 

In a subsequent study, the same group regenerated cellulose fibers from TBAAcO and DMSO without 18-crown-6. They used a softwood cellulose (DP = 1050) and prepared solutions of 4–10 wt % at 40 °C. The resulting fibers had circular cross-sections and smooth surfaces. Analyses of DP after regeneration indicated no detectable degradation of the regenerated cellulose. The fibers showed tenacities between 2–3 cN/dtex and elongation values between 9.7–11.8% [121]. Thus, the dry strength values are similar to those of viscose (20–25 cN/tex, 18–23% elongation) or cotton (24–36 cN/tex, 7–9% elongation) [174].

The use of a recycled TBAAcO/DMSO solvent for the dissolution and regeneration of cellulose was recently investigated [175]. It was shown that the TBAAcO/DMSO solvent system tolerates addition of some non-solvents, such as water or ethanol, without cellulose precipitation. Mechanical properties of spun filaments were not altered when prepared from a simulated recycled solvent containing 2 wt % (cellulose) non-solvent. The ability of the system to tolerate non-solvents was dependent on the concentration of both cellulose and TBAAcO. By increasing the concentration of TBAAcO, more water can be tolerated for the same cellulose content [111]. 

Carbon nanotubes were added to a solution of cellulose in TBAAcO/DMSO, and were then formed into composite fibers, spun at room temperature and then coagulated in a water bath. Characterization of the fibers using SEM showed that the fibers were smooth and did not contain micro pores, which indicated that the carbon nanotubes were well-dispersed in the cellulose matrix. It was also shown that the mechanical and thermal properties of the composite fibers (5 wt % carbon nanotubes) compared to neat cellulose fibers were improved: increased tensile strength (approx. 20%), elongation at break (approx. 20% increase), and thermal stability (decomposition temperature increased by ca 20 °C) [176].

The TBAAcO/DMSO system was used for the homogeneous esterification of cellulose without catalysts. After 5 h at 60 °C using a 5 molar excess of acetic anhydride per AGU an organo-soluble cellulose acetate with a DS_Ac_ of 2.91 was reached. Under the same conditions, cellulose propionate with a DS_Pr_ of 1.83 was obtained. Cellulose acetate/propionate and acetate/butyrate mixed esters with almost full functionalization were obtained [177,178]. The conversion of cellulose with succinic anhydride under catalyst free conditions yielded products with DS_Succinate_ between 0.3 and 1.2 after 1 h at 60 °C. As known, these products successfully absorb heavy metal ions such as copper(II) and cadmium(II) from aqueous solutions. The concentration of absorbed metal ions increased as a function of increasing DS_Succinate_ [179]. 

A binary mixture of tetrabutylammonium propionate with methylimidazole (2.5/7.5 by weight) dissolves 15.7 wt % MCC at 25 °C. The main driving force of the dissolution was attributed to the propionate anion, while methylimidazole was mainly acting as solvent, promoting dissociation of the ions. The authors also claim that the architecture of regenerated cellulose films can be adjusted in dependence of the processing strategy [180]. A set of 20 tetra-(*n*-alkyl)ammonium acetates and propionates was synthesized and studied regarding the influence of the cation on cellulose dissolution, vide 3.1.2. The majority of these QAE were able to dissolve cellulose with and without the addition of DMSO, and triethylhexylammonium acetate was the best QAE in the set studied; 22 wt % cellulose at 90 °C [73].

Neat *N,N*-allylmethylmorpholinium acetate was shown to be an efficient cellulose solvent. Thus 17, 28, and 30 wt % MCC were soluble in this QAE at 80, 100, and 120 °C, respectively, even in the presence of some water. Also 25 wt % of high molecular weight samples (DP = 2082) was dissolved without noticeable degradation, as confirmed by SEC analysis. SEM micrographs of native and regenerated cellulose indicated that upon regeneration, the surface of cellulose fibers became smoother, and uniform, as compared to rough and scattered surface of untreated cellulosic fibers. This was attributed to re-aggregation of strongly bonded crystalline cellulose fibers into more homogeneous macromolecular assembly upon dissolution [181].

The following QAEs for cellulose dissolution and processing are based partially on naturally occurring compounds. Ohira et al. prepared QAEs with amino acid moieties as anions. *N*,*N*-Diethyl-*N*-(2-methoxyethyl)-*N*-methylammonium alaninate (N_122 Me(OEt)_) was the best cellulose solvent for MCC, dissolving 6 wt % at 60 °C and 12 wt % at 100 °C within 10 minutes. The corresponding acetate and chloride dissolved 7 and 10 wt % MCC, respectively, although much more slowly, ca. 48 h for complete dissolution. The fact that the amino acid anions are better than the corresponding carboxylates may be due to the extra amino moiety present in the former anion. Tryptophanate, lysinate, and threoninate were tested as well; they dissolve MCC between 1 and 11 wt%. When DMSO was added to *N*,*N*-diethyl-*N*-(2-methoxyethyl)-*N*-methylammonium alaninate (1:1 mixture by volume, which equals a molar fraction of DMSO χ**_DMSO_** = 0.75) 11 wt % MCC were soluble at 25 °C after 10 min, and 22 wt % after 6 h, or after 10 minutes at 100 °C [182,183]. The dissolution of cellulose in these solvents seems to be more suitable for fiber spinning compared to commonly used IL [22]. Cholinium cation-based QAEs with 28 amino acid and carboxylate anions were used for dissolution of biomass [184]. The solubility of cellulose in these QAEs was poor (<1 wt % in dicholinium malinate), but they dissolve lignin readily. They were used, therefore, for delignification of rice straw, which confirmed similar results reported earlier [185]. 

**Figure 10:**
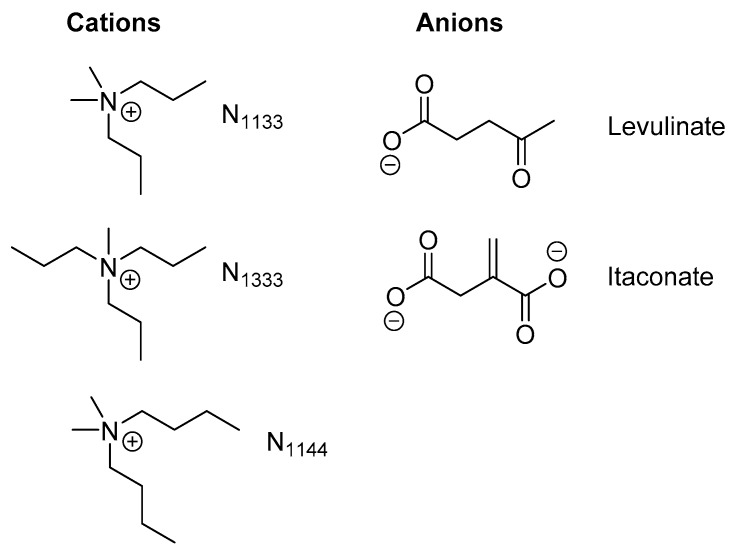
Structure of the levulinate and itaconate-based QAE used for cellulose dissolution [186].

Various tetra-(*n*-alkyl)ammonium cations (N_1133_, N_1333_, N_1144_) were combined with the levulinate anion (Figure 10). The cellulose solvent obtained dissolved 10 wt % MCC in the neat N**_1333_** levulinate. Moreover, this cellulose solution exhibited exceptional tolerance towards water. For example, this QAE could still dissolve cellulose efficiently in the presence of 18 wt % water, an improvement of potential industrial significance because the energy consuming drying of the substances is not required. By addition of 20 wt % γ-valerolactone, a co-solvent based on renewable resources, the cellulose solubility increased to 20 wt % [186].

Relevant examples of carboxylate-based QAEs as cellulose solvents are summarized in Table 2.

### 4.3. Quaternary Ammonium Halides

#### 4.3.1. Quaternary Ammonium Chlorides (QACls)

**Figure 11:**
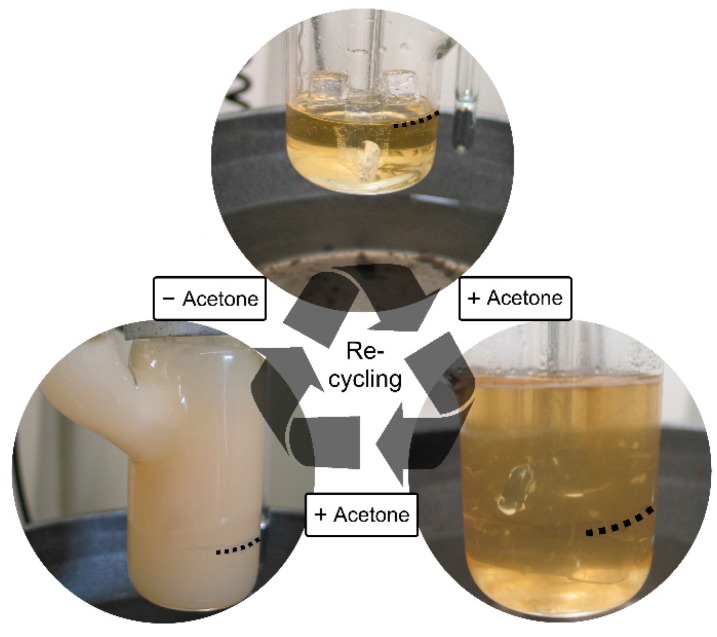
Recycling scheme of a regeneration-dissolution cycle of cellulose in QAE/acetone by simple addition or removal of acetone; reprinted with permission from [195], Copyright (2015) GIT Labor-Fachzeitschrift, Wiley-VCH Verlag.

Until present, only a few QACls, which dissolve cellulose, were used for various derivatization reactions. Benzyldimethyltetradecylammonium chloride (N_11-14-Bz_ Cl) dissolves up to 5 wt % MCC, a cellulose concentration that is sufficient for chemical modification of the biopolymer but not high enough for its shaping [187]. N_11-14-Bz_ Cl was used for the homogeneous etherification of cellulose to obtain hydroxypropyl cellulose [188]. This compound belongs to the group of “benzalkonium chlorides” and is commercially available as trihydrate or in solution as disinfectant or textile softner. Other QACls that dissolve MCC are triethylheptylammonium chloride, N_2227_Cl (10 wt%), and triethyloctylammonium chloride, N_2228_Cl (15 wt%) [189,190,191,192,193]. Several co-solvents were employed as well in order to decrease the viscosity and avoid high dissolution temperatures, in particular DMAc and DMSO. For example, up to 10 wt % MCC dissolves at 60 °C in 1:1 N_2228_Cl/DMAc mixtures by weight; the N_2228_Cl concentration can be decreased by further addition of DMAc until the mole fraction (χ) was the same as in a conventional LiCl/DMAc mixture (χ_N2228Cl_ =χ_LiCl_) without affecting the ability to dissolve cellulose. The DMAc/N_2228_Cl solvent was successfully used as SEC eluent for the determination of cellulose molar mass distribution [61]. Besides DMAc, DMF, DMI, pyridine and, surprisingly, acetone were found to form translucent cellulose solutions of low viscosity. Because of its volatility, acetone is a specially promising diluent for cellulose shaping (Figure 11). ^13^C-NMR spectra of cellulose dissolved in N_2228_Cl/DMSO and N_2228_Cl/acetone revealed that no derivatization occurred on the cellulose backbone [192,193]. In subsequent studies it was found that solutions of N_2228_Cl in the following MSs dissolve cellulose: DMSO, DMAc, DMF, 2-butanone, *N*-methyl-2-pyrrolidone (NMP), 1-methylimidazole, ethyl acetate, 2-methyltetrahydrofuran, tetrahydropyran, 1,3-dimethyl-2-imidazolidinone, and 3-pentanone [194]. These cellulose solutions exhibited comparably low viscosities, which is beneficial for derivatization reactions but presumably disadvantageous for fiber spinning (Table 3).

Studies on cellulose derivatization were carried out [194,196]. Silylation, tosylation, carbanilation and especially acetylation reactions were conducted successfully in the binary mixtures of N_2228_Cl/MS. The latter one led to cellulose acetates with DS_Ac_ ranging from 0.16 to 2.79 depending on the reaction conditions. Within 2 h at 50 °C using acetyl chloride/pyridine an organo- soluble (acetone, DMSO, CHCl_3_) cellulose acetate with DS_Ac_ = 2.79 was isolated. Under the same reaction conditions but with LiCl/DMAc or BuMeImCl as solvents the DS_Ac_ values were almost identical with 2.83 and 2.81.

A mixture containing tetraethylammonium chloride was used for cellulose dissolution. The optimum conditions to dissolve up to 8.7 wt % cellulose within 15 min were a mixture of: NaOH 8 wt%, DMAc 10 wt%, N_2222_Cl 6 wt%, water 76 wt % and a dissolution temperature of −5 °C. No degradation or derivatization during dissolution or regeneration was observed [197].

#### 4.3.2. Quaternary Ammonium Fluorides (QAFs)

**Figure 12:**
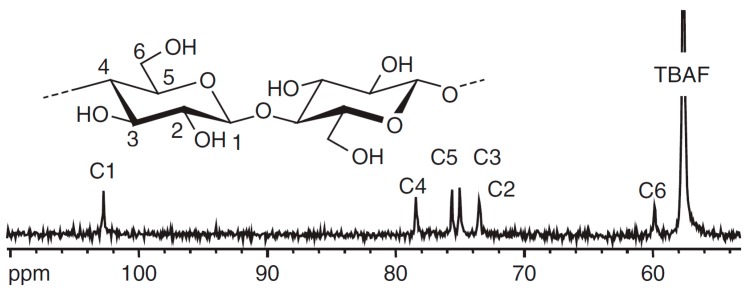
**^13^**C-NMR spectrum of unmodified cellulose dissolved in dimethyl sulfoxide-d*_6_*/TBAF·3H_2_O, reprinted with permission from [16], Copyright (2012) Elsevier.

Among QAFs, the cellulose solvent most investigated is the mixture DMSO/TBAF·3H_2_O, first described as solvent for cellulose in 2000. This solvent enabled cellulose dissolution with a degree of polymerization up to 650 within 15 min without pretreatment, e.g., previous drying. The ^13^C-NMR spectrum showed that DMSO/TBAF·3H_2_O belongs to the class of non-derivatizing solvents, as confirmed by the presence of the signals of unmodified AGU (Figure 12) [173].

DMSO/TBAF·3H_2_O was used for homogeneous derivatization of cellulose by transesterification employing vinyl esters of different carboxylic acids, resulting in cellulose esters with varying DS. Ciacco et al. employed sisal cellulose for reactions with acetic anhydride, stearic anhydride, vinyl acetate and vinyl laurate. The DS-values of the esters produced showed that the anhydrides were less reactive as compared to the vinyl esters. This observation may be attributed to the much faster hydrolysis of the anhydrides (relative to the vinyl esters) by water introduced by TBAF·3H_2_O. This conclusion was corroborated by the observation that the obtained DS_Ac_ was 1.29 and 0.3 for experiments run under identical experimental conditions except for TBAF·3H_2_O of 6 and 11 wt%, respectively. Because the use of molecular sieves proved inefficient, and use of NaH led to the formation of dimsyl ions and side reactions, water was removed by distillation of 30 vol% DMSO [198]. This strategy is akin to water removal from the system cellulose/LiCl-DMAc [199]. The partial drying of TBAF·xH_2_O/DMSO increased the DS_Ac_ to 1.15. The distillation of 50 vol% of DMSO before acetylation of cotton linters resulted in an increasing DS_Ac_ from 1.0 to 1.8 [200]. As discussed above, dry TBAF can be generated *in situ* from the reaction of TBACN with C_6_F_6_. Although the solvent was successfully employed for cellulose acylation and carbanilation, it contains hexacyanobenzene byproduct that forms radical anions readily [201,202]; cellulose was insoluble in this medium after 28 h [93]. Regarding the molecular structure of the acylating agent, it was observed that the DS-values of the ester increased with increasing chain length of the acylation agents. As shown from kinetic data of cellulose acylation by carboxylic acid anhydrides in LiCl/DMAc and IL/MSs, the reason for this dependence is a compensation of the reaction entropy and enthalpy of activation [88,203].

**Figure 13:**
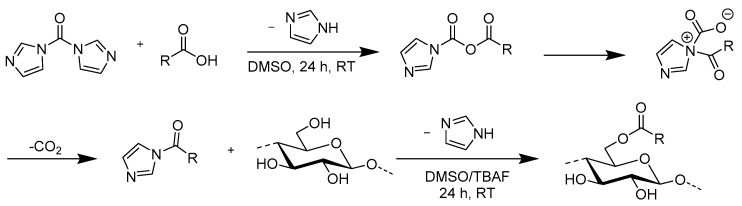
Schematic demonstration of CDI activation of carboxylic acids; redrawn from [141].

Cellulose esters were synthesized in TBAF·3H_2_O/DMSO using the following carboxylic acids: acetic, stearic, adamantane-1-carboxylic, and 2-furancarboxylic, in the presence of *N*,*N*′-carbonyl- diimidazole (CDI) as acid activation agent, see the reaction scheme in Figure 13 [204].

Subsequent studies used the same activation approach with CDI in TBAF·3H_2_O/DMSO to produce cellulose esters with elaborate structures. The acids employed included (−)-menthyloxyacetic acid, 3-(2-furyl)-acrylcarboxylic acid, furan-2-carboxylic acid and 4’-carboxybenzo-18-crown-6 as well as carboxymethyl-β-cyclodextrin [205]. Another activating agent, 1*H*-benzotriazole, was employed with the following carboxylic acids: acetic, benzoic, butyric, caprylic, myristic and stearic. Use of this activating agent in TBAF·3H_2_O/DMSO led to cellulose esters, whereby the preferred functionalization occurred at C-6 of the AGU [206]. 

**Figure 14:**
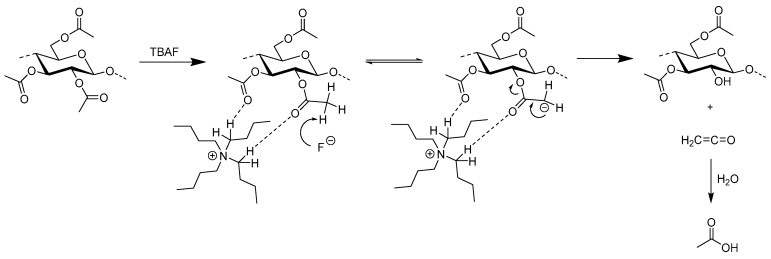
Proposed deacetylation mechanism of cellulose in the presence of TBAF; redrawn from [207,209].

Another interesting application of TBAF·3H_2_O/DMSO is regioselective deacetylation. During deprotection of silylated cellulose acetate with TBAF·3H_2_O/THF, cleavage of the acetate groups was observed. Further investigations employing cellulose acetate revealed regioselective deacetylation at positions C-2 and C-3 of the AGU [207,208]. The regioselectivity of deacylation was explained by the mechanism shown in Figure 14, where TBA^+^ acts as a general-acid catalyst, whereas F^−^ induces deacetylation via ketene intermediate [209]. Formation of the latter was inferred from a kinetic isotope effects experiment [210].

Formation of cellulose succinate was investigated in the presence of N_2222_Cl/DMSO and TBAF·3H_2_O/DMSO. The resulting DS_Succinate_ increased with increasing concentration of the respective QAE until 11 wt%, DS = 1.88 and 2.09 were obtained, respectively. Increasing the QAE concentration to 16 wt % increased the DS_Succinate_ to 2.01 for N_2222_Cl/DMSO, but resulted in decrease in case of TBAF·3H_2_O/DMSO. The results of intrinsic viscosity of a solution of Mulberry wood cellulose and 2D NOESY experiments of cellobiose in both solvents were explained on basis of formation of hydrogen bonds between the hydroxyl groups (of AGU or cellobiose) and the relatively acidic α-methylene hydrogens of N_2222_^+^ or TBA^+^.

Thus, the concentration of QAE, and steric hindrance by QAE^+^ control the accessibility of the hydroxyl groups of the AGU, affecting the DS_Succinate_-value [211]. The production of superabsorbent hydrogels was carried out using succinic anhydride in LiCl/NMP and TBAF·3H_2_O/DMSO in the presence of DMAP as catalyst. Esters with similar DS_Succinate_ between 0.1–3.0 were obtained in both solvents, depending on the reaction conditions. Solution gelation was observed during this synthesis; it was attributed to crosslinking, initiated by DMAP. The products obtained at 60 °C showed similar or even better water absorbency compared to the “conventional” absorbent sodium polyacrylate [212]. Cellulose derivatization by bulky acyl moieties to produce precursors for dendronized cellulose derivatives was carried out in TBAF·3H_2_O/DMSO and LiCl/DMAc. Thus, 3,5-bis(benzyloxy)benzoic acid (activated with CDI), and 3,5-bis(benzyloxy)benzoyl chloride reacted with cellulose. Structure elucidation of the esters by means of FTIR and NMR indicated substitution at position C2 despite the bulkiness of the substituents [213].

Besides acylation, cellulose etherification was carried out in TBAF·3H_2_O/DMSO, e.g., sisal cellulose and cotton linters was modified using benzyl chloride in the presence of finely divided solid NaOH or aqueous NaOH solution. The DS_Bz_ varied between 0.4 and 2.85; a large excess of NaOH (NaOH/AGU molar ratio 6/1 to 18/1) was needed to obtain products with a higher DS_Bz_. The reactions employed without NaOH led to low DS-values, indicating that the basicity of the F^−^ ion alone is not sufficient for activating the hydroxyl groups of the polymer [214]. The structure of cellulose benzyl ether and hence its solubility in MS (e.g. DMSO, DMAc, DMF) depended on the chosen reaction conditions, either the homogeneous reaction in TBAF·3H_2_O/DMSO, or the heterogeneous one in aqueous NaOH. ^1^H-NMR data indicated that benzyl moieties are regularly distributed along the polymer backbone in the product from the homogeneous reaction, leading to better solubility, whereas a block-like distribution was found in case of the heterogeneous reaction [215].

Another etherification of cellulose studied was the synthesis of CMC, which is of great industrial interest, regarding its application as thickener. Successful modification of cellulose employing sodium monochloroacetate in the presence of NaOH (powder suspended in DMSO) yielded CMC with DS-values from 1.82 to 2.09 within 1 h. More detailed investigations of CMC preparation from mercerized sisal cellulose and cotton linters examined the effects of different reaction conditions [214]. The influence of solid or aqueous NaOH on the resulting substitution patterns was studied. The use of NaOH powder led to higher reactivity in TBAF·3H_2_O/DMSO, but samples that are less soluble in water, due to deviation from the conventional statistical substitution pattern of the carboxymethyl groups [216].

The synthesis of allyl cellulose in TBAF·3H_2_O/DMSO is interesting because the produced ether can be further modified by addition to the double bond. Allyl cellulose with DS_Al_ from 0.5 to 2.98 were obtained [217]. Additionally, TBAF·3H_2_O/DMSO was also employed for unconventional cellulose products. Thus, graft polymerizations on cellulose were performed in TBAF·3H_2_O/DMSO employing lactones or *N*-carboxy-α-amino acid anhydrides [218]. TBAF·3H_2_O/DMSO was employed for the synthesis of 6-deoxy-6-fluoro cellulose derivatives. Here the QAF is acting as solvent and reagent for converting cellulose tosylate with a DS_Tosylate_ of 1.22 to 1.74 into deoxy-fluoro cellulose with DS_F_− up to 0.41. Compared with other cellulose deoxy-halides, the fluorination could be performed at room temperature. Variation of the water content of TBAF·xH_2_O, obtained by distillation of DMSO, exhibited no correlation between the DS_F_ and the amount of water removed [219].

The applications of QAFs, other than TBAF·3H_2_O, are less described in literature. However, another cellulose solvent is tetraallylammonium fluoride monohydrate (TAAF·H_2_O)/DMSO. Homogeneous acetylation of different types of celluloses (MCC, cotton, eucalyptus) were performed in this solvent, resulting in CAs with DS_Ac_ from 0.4 up to 2.4, depending on reaction conditions. An interesting result was observed for prolonged reaction times. Despite the use of an excess acetylation agent, the resulting DS_Ac_ were relatively low, which was attributed to the fluoride ion mediated ester hydrolysis. The latter conclusion was corroborated by following the change of DS_Ac_ of a commercial CA sample (DS_Ac_ = 2.7) as a function of time, at 60 °C, in absence of acetic anhydride; DS_Ac_ decreased to 1.7 and 1.1 after 6 and 18 h, respectively. An interesting point that is relevant to all acylation reactions with reactive carboxylic acid derivatives (anhydrides and acyl chlorides) is the formation of acetyl fluoride from TAAF·H_2_O and acetic anhydride, as indicated by FTIR. Therefore, it is possible that many esters are produced by the reaction of cellulose with two acylating agents, namely carboxylic acid anhydride (or acyl chloride) and the corresponding carboxylic acid fluoride [109].

One problem with TAAF·H_2_O is its polymerization tendency. To suppress this side reaction, Casarano et al. used dibenzyldimethylammonium fluoride/DMSO with much less water content (Bz_2_Me_2_AF·0.1H_2_O) [110]. This QAE/DMSO solution is efficient in cellulose dissolution and acylation. Based on molecular dynamic simulations, the superior properties of BzMeAF·0.1H_2_O, compared to TBAF·3H_2_O, were attributed to the more desolvated fluoride anion which leads to stronger interactions (Cel-OH···F^−^), hence better cellulose dissolution capacity and higher biopolymer reactivity. The same solvent mixture was also employed for etherification of cellulose. Therefore allylation, benzylation and carboxymethylation of different types of cellulose (MCC, cotton) were carried out. The reactions were performed following the same conditions described for TBAF·3H_2_O/DMSO. The resulting DS-values of the etherified celluloses in Bz_2_Me_2_AF·0.1H_2_O/DMSO were comparable to those obtained in TBAF·3H_2_O/DMSO [90].

Table 4 shows selected results of dissolution experiments of cellulose in the discussed QA halides. For the last 3 entries the amount of dissolved cellulose is given as used for the esterification reactions. The maximum amount of cellulose soluble in these mixtures was not determined.

### 4.4. Other Ammonium Electrolytes and Deep Eutectic Solvents (DES)

Tetraalkylammonium dimethylphosphates have already been patented as solvents for cellulose and reaction media for the production of cellulose esters [220,221,222]. Tri-(*n*-butyl)methylammonium dimethylphosphate (TBMA DMPh) dissolved 12.5 wt % MCC and allowed the homogeneous, catalyst-free synthesis of cellulose acetate with a DS_Ac_ of 2.5 using only 3 moles of acetic anhydride/mole AGU in less than 30 min in the neat QAE. DMSO, DMF and NMP were efficiently used as co-solvents to obtain cellulose acetate and cellulose acetate/propionate mixed esters. Cellulose can be dissolved in (2-hydroxyethyl)trimethylammonium- (6.5 wt%), (2-ethoxyethyl)trimethylammonium- (5.9 wt%), tri-(*n*-pentyl)methylammonium- (2.1 wt%) and trimethylethylammonium dimethylphosphate (1.0 wt%) [222].

The concept of DES goes back to a publication of Abbott et al. [223]. These ionic fluids are obtained by mixing hydrogen bond acceptors, e.g. choline chloride (ChCl), with hydrogen bond donors, e.g., amides such as urea. In principle, they can be viewed as a type of mixed ILs, too, but since they do not consist exclusively of ions the term DES was introduced to distinguish between both classes of fluids [224]. In principle, they share the advantages of ILs, such as non-volatility and structure versatility, but overcome some of their disadvantages, e.g. problematic purification or limited biocompatibility with enzymes [225]. Recently, comprehensive reviews covering this field were published [226,227]. Therefore, we only focus on studies regarding cellulose. 

Up to 6.5 wt % cotton linters was soluble in a mixture of allyltriethylammonium chloride and oxalic acid at 110 °C after 2 h [228], which is one of the highest values reported for DES. Only 2.5 wt % was soluble in ChCl/imidazole [229]. ChCl/lactic acid (1/10) dissolved ca. 3 wt % cellulose and 13 wt % lignin. These differences in the capability of dissolving biomass constituents open up a promising alternative towards new delignified cellulosic material [230]. Generally, DES dissolve lignin significantly better than cellulose. Since DES possess strong hydrogen bonds, the amount of free chloride ions is lower than in QAE or ILs, which may be the reason for lower cellulose dissolution capacity, as shown in Table 5.

The acidic treatment of wood cellulose fibers with ChCl/oxalic acid led to the degradation of the amorphous regions without affecting the crystalline domains. Subsequent disintegration yielded cellulose nanocrystals with a width/length ratios of 9–17/310–410 nm. Compared to common procedures for obtaining nanocrystals, this method uses milder conditions with easily obtainable and biodegradable solvents [231]. Treatment of cellulose board pulp grades with ChCl/urea showed enhanced nanofibrillation. This behavior is a promising route to treat waste board and paper [232]. 

There are some examples for the successful derivatization of cellulose in DES. Thus, Abbott et al. carried out acetylation reactions with monosaccharides and cellulose. ChCl/ZnCl_2_ was used as reaction medium, acetic anhydride as reagent and the mixture was stirred for 3 h at 90 °C. DS_Ac_ between 0.4 and 1.5 were obtained. The partial solubility of the samples in acetone indicates a block like distribution of the acetyl substituent along the polymer chain. This result is typical for reactions conducted under heterogeneous conditions (swollen solid cellulose/acetylating bath). Therefore, it is assumed that the cellulose used in this experiment was suspended or just partially dissolved in the DES [233]. Etherification reactions towards cationic functionalized cellulose for potential treatment of waste water of the textile industry were reported, using chlorocholine chloride/urea solvent [234]. After 15 h at 90 °C in presence of NaOH, ethyltrimethylammonium cellulose with a substitution of 0.5% of quaternary nitrogen per mole of AGU was isolated, which corresponds to a DS < 0.1. Other modification reactions of cellulose in DES were studied, leading to cellulose succinylates and methylcarbamates. The solvents employed were LiCl/urea and ZnCl_2_/dimethylurea, respectively [235,236].

## 5. Salts of Superbases

As indicated in Section 3.2, a new class of cellulose solvents are salts of superbases [142]. The cellulose dissolution capacity of the different salts varies. Several acid-base conjugates were prepared by combining bases and superbases with acetic and propionic acid [143]. For TMG, short-chain carboxylates were able to rapidly dissolve 5 wt % MCC in less than 10 min. The formates and butyrates needed longer to dissolve the same amount of cellulose. Cellulose regenerated from TMGAcO solution exhibited slightly lower molecular weights than the starting material, which was attributed to the presence of small amounts of excess acetic acid. The occurrence of polymer degradation in carboxylate-based ILs is not unusual as the authors showed in comparative dissolution experiments with the IL EtMeImAcO [142]. A comparison of the dissolution capacities was also made between the propionates of TMG, DBN and DBU. High molecular weight cellulose (PHK) pulp was used and the maximum biopolymer loading was compared with EtMeImAcO; both solvents have comparable dissolution efficiency, 15–16 wt % and 18 wt % for the QAEs and IL, respectively. A significant advantage of these QAEs is that they are, *unlike ILs*, distillable under reduced pressure, i.e., the pure base can be recovered and reused [143].

The salts of super bases were employed as reaction media for cellulose derivatization, e.g., acetylation under homogeneous conditions. The derivatization was carried out with acetic anhydride in combination with different MSs (acetone, acetonitrile, DMSO). DS_Ac_ values in the range from 0.90 to 2.89 were obtained depending on the chosen reaction conditions [237]. TMG-based QAEs, including TMG tetrafluoroborate and TMG lactate, were employed for conversion of MCC to 5-hydroxymethylfurfural. In this application, DMAc/LiCl was the solvent, whereas the QAE acted as catalyst [238]. TMG was also employed to obtain cellulose gels. The latter were produced by combining cellulose at room temperature with TMG in different ratios and were evaluated for possible applications to capture CO_2_ [239].

**Figure 15:**
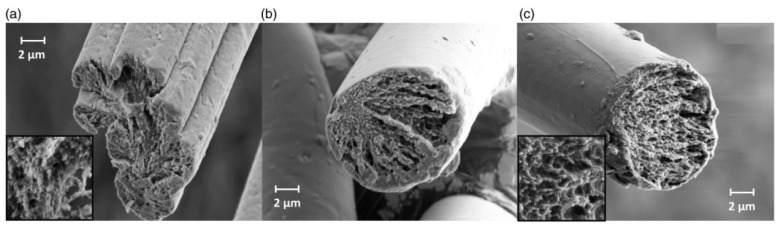
Scanning electron microscopy images of viscose (**a**), Lyocell (**b**) and Ioncell-F (**c**). The insets in the left corners show a 2.8 and 2.5 times magnification of the body of the viscose (**a**) and Ioncell-F (**c**); reprinted with permission from [11], Copyright (2016) by SAGE Publications.

There are also interesting developments in the use of salts of super bases for shaping and regeneration of cellulose, e.g., for production of cellulose textile fibers or as reinforcing structures in composite materials [240]. The process suggested, called Ioncell-F, is based on DBN AcO as solvent for cellulose. Although DBN AcO is solid at room temperature, its low melting point (63 °C) and the low viscosity of the melt makes it a useful medium. The fiber spinning technique is a dry-jet wet spinning process where the cellulose solution (15–17 wt%) is extruded and drawn in air and the fibers become highly oriented upon coagulation in cold water, resulting in fibers with high tenacities. The morphology of the fibers is similar to the Lyocell fibers, with smooth fiber surfaces, round cross-sections, and homogeneous, dense fibrillar structure (Figure 15) [11]. An advantage of the process is also that moderate process temperatures of 75–80 °C is used, which avoids the otherwise extensive cellulose degradation.

The Ioncell-F process has been covered in a series of publications, and the earlier studies were conducted with a simple experimental spinning set-up, using a monofilament spinneret with a diameter of 100 μm [241,242]. It was found that the spin dope had similar rheological properties as the NMMO dope. An investigation was conducted with focus on the effects of the spinning parameters on the mechanical properties and orientation of the fibers. The parameters investigated included spinning stability, extrusion velocity, draw ratio, spinneret aspect ratio, and coagulation bath temperature (for definition of these terms see the Supplementary Material). It was found that mechanical properties were independent of extrusion velocity and that the orientation of the fibers was nearly complete at a draw ratio of 5. Another conclusion was that fibers with high tenacities were formed at 15 °C (coagulation bath), but not at 30 or 45 °C. In following studies, the spinning equipment was upgraded to a multihole spinneret with 36 holes and a capillary diameter of 100 μm [243]. The spinning process was evaluated using a 13 wt % dissolved PHK pulp. Results of molar mass distribution before and after cellulose dissolution (at 80 ^ο^C) and regeneration showed a slight degradation of the high molecular weight fraction. This degradation, however, was substantially lower than that reported for imidazolium-based ILs or NMMO. Additionally, it was found that the draw ratio can be gradually increased up to 18, which led to fast increase in fiber orientation and crystallinity. Tenacity values similar to those of commercial Lyocell fibers (40–42 cN/tex) were reached at medium draw ratio of roughly 5, and further increased draw led to fibers with tenacities above 50 cN/tex. Fibers from this process were also used for yarn spinning, and compared to commercial viscose fibers, using the same equipment. DBN AcO based yarns had higher tenacity but lower elongation and the yarn prepared from the Ioncell-F fibers was also slightly thinner and more uneven than the viscose yarn. Furthermore it was shown that enhanced spinnability increased orientation and high-tenacity fibers were achieved when the cellulose solutions had a large (>20%) fraction of high molecular weight cellulose (DP > 2000) and a minimum polydispersity index of 3.4 [244].

Using the Ioncell-F process for textile applications was further investigated [11]. Ioncell fibers were compared with commercial viscose fibers and converted into two-ply yarn using ring spinning technology. The yarns made using DBN AcO were stronger but more brittle and had higher irregularities than the viscose yarns. However, the Ioncell yarns showed good behavior during the knitting and weaving processes and were produced into garment demonstrators, such as a knitted scarf and a dress. The Ioncell yarn could be dyed in a batch process with reactive dyes that are commonly used on industrial scale. Detailed studies on reasons to spinning failure, such as a breach in the coagulation bath, has also been covered [245]. It was found that NMMO·2H_2_O and DBN AcO were good spinning solvents, while EtMeImAcO and TMG AcO were poor spinning solvents. The extent of stretching of the forming filaments was simulated by calculating the diffusion dynamics. Thus a good solvent for spinning must also be a solvent that solidifies the structure of the fiber during regeneration, thus permitting increased and retained orientation. Gelatinous dopes that already have a gel network before regeneration or dopes that weaken upon the addition of water (water progressing towards the center of the fiber and leading to fiber breach) were instead poor spinning solvents.

Moreover, the Ioncell-F process has been investigated with cellulose from other types of feedstock. The use of cotton waste to produce virgin fibers of higher quality compared to mechanically recycled material has been reported [246]. However, it was necessary to use a pretreatment to adjust the DP of the cellulose. The material was dissolved and regenerated, and fibers with tenacities as high as 58 cN/tex (870 MPa) were obtained. Another approach the use cellulosic waste, e.g., paper and cardboard, and solubilize all biopolymers in the material (i.e. also hemicelluloses and lignin) in the DBN AcO solvent to prepare a spin dope [247]. Also in this case, a series of refining steps (mechanical, chemical, enzymatic) of the raw material and an adjustment of the DP of the cellulose were required to achieve dissolution. The fibers showed again high tenacities and prototype textiles were produced to evaluate the fiber quality and the possibility to use lignin as a natural (beige/brown) dye. 

A challenge to reach commercial success for the Ioncell-F process, and in general for ILs and QAEs, is the development of a viable and efficient solvent recovery system. Initial laboratory scale recycling trials were conducted and the results showed that DBN AcO can be recycled from aqueous media with an average recovery rate of 95.6 wt % using rotary evaporators [248]. The recycling of the solvent did not affect the chemical composition or DP of the recovered cellulose, however the color of the regenerated material became gradually darker with each regeneration cycle. Additionally, DBN AcO itself underwent detectable hydrolysis (6.0–13.6 mol% per cycle) yielding 3-(aminopropyl)-2-pyrrolidonium acetate. With increasing amount of this byproduct solubility of cellulose decreased and ceased at 30.6–45.6 wt % hydrolysis product. Water evaporation using a thin film short evaporation path; stability and toxicity tests of DBN AcO, and tests to remove oligosaccharides from the coagulation bath are being conducted [247]. 

**Figure 16:**
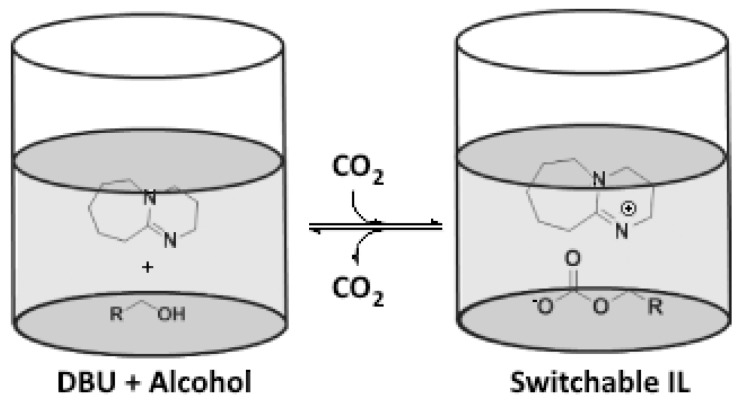
Illustration of the concept and mechanism of switchable ILs; reprinted with permission from [226], Copyright (2013) Society of Chemical Industry.

Switchable ILs are another class of electrolytes worth a short mentioning in the frame of this review [226]. They are formed by mixing equimolar amounts of an alcohol with a strong organic base, e.g. amidines such as DBU, with gaseous CO_2_ at ambient pressure and room temperature. By combining these three components, an exothermic transformation takes place, converting the mixture to an ionic fluid by formation of the alkyl carbonate, between CO_2_ and the alcohol to subsequently form the amine salt (Figure 16). Addition of N_2_ or other gases leads to a shift of the CO_2_, and the system is surprisingly found to be fully reversible, resulting once more in the starting materials [249]. Suitable applications for such QAEs are supposedly in biomass processing [250,251,252] or for CO_2_ capture [253]. The switchable IL that is produced from DBU, methanol and CO_2_ was also shown to be able to dissolve cellulose. It could be used as reaction media for acylation of cellulose and yielded DS_Ac_ values of up to 2.94 depending on reaction conditions [254].

## 6. Conclusions

Solutions of QAEs in MSs are exciting solvents for cellulose because of their structural versatility, and easy recycling. Cellulose dissolution is favored if the QAE is composed of a small, hard anion and a voluminous cation. The dissolution proceeds by a cooperative mechanism. The anion interacts with the hydroxyl groups of the AGU, leading to hydrogen bond disruption; the polymer chain thus acquires a negative charge. The repulsion between the negatively charged cellulose-anion complex is enhanced by: (i) steric crowding due to the attached sheath of the corresponding cation of the QAE; (ii) solvophobic interactions of the latter with the hydrophobic face of the AGU. Thus, the hardness of the anion and the hydrophobic character of the cation are important for cellulose dissolution. 

The most employed QAEs are the halides, in particular TBAF·3H_2_O, the carboxylates, hydroxides and dialkyl phosphates. Solutions of cellulose/QAE/MS were successfully employed for derivatization of cellulose under homogeneous conditions into esters and mixed esters. Cellulose ethers were also obtained under homogeneous and heterogeneous conditions with difference in the distribution of the ether group along the biopolymer backbone, leading to different solubility, e.g., in water. Solutions of QA-hydroxides are suitable for cellulose dissolution, shaping and derivatization if the reagent is not particularly sensitive to hydrolysis. 

The use of QAEs for regeneration and shaping as well as chemical modification of cellulose was reported to a limited extent in the literature. However, initial results from cellulose dissolved in QAEs, dominantly TBAAcO and TBAOH, showed high potential and their use in processing of cellulose is an active field of investigation. QA halides, such as TBAF and N_2228_Cl, proved to be excellent media for various acylation reactions. Some unconventional products are reported as well. Dissolution of cellulose in salts of super bases revealed promising results, and there are currently intensive research on shaping cellulose from such solvents. The formed fibers (Ioncell-F) showed favorable mechanical properties. However, the recovery of the solvent from such processes needs to be developed further. A common protocol for cellulose dissolution is needed to provide more consistency in data reporting, as already mentioned by Wang et al. [138] and Pinkert et al. [18,255]. 

The intended application of each cellulose solution, e.g., fiber spinning or chemical modification is a decisive factor for the choice of the right solvent system. From the sustainability point of view, the solvent should be stable under processing conditions and easily to recycle in a continuous process. The importance of other properties such as viscosity of the biopolymer solution and toxicity of QAE and MS should be taken into consideration.

## Figures and Tables

**Table 1:** Examples of the solubility of cellulose in tetra-(*n*-alkyl)ammonium hydroxide aqueous solutions.

Cellulose Solvent	Dissolved Cellulose [wt%]	Dissolution Conditions	Cellulose Type (DP) ^2^	Reference
TMAOH_aq_ (30 wt%)	swelling	RT	- ^1^	[162]
TMAOH_aq_ (25 wt%)	10	RT, 2 min	MCC	[165]
TBAOH_aq_ (40 wt%)	10	RT, 24 h	MCC	[119]
TBAOH_aq_ (40 wt%) + 18-crown-6 (2.0 M)	10	RT, 10 min	MCC	[119]
TBAOH_aq_ (55 wt%)	10	RT, 2 min	MCC	[164]
TBAOH/urea/H_2_O (33/17/50 wt%)	7	RT, 1 h	Softwood kraft pulp (660), MCC	[154]
TBAOH/DMSO/H_2_O (12/70/18 wt%)	6	- ^1^	PHK pulp (630)	[166]

^1^ - No data given, ^2^ DP - average degree of polymerization in brackets, where available

**Table 2:** Examples of the solubility of cellulose in quaternary ammonium carboxylates.

Cellulose Solvent	Dissolved Cellulose [wt%]	Dissolution Conditions		Cellulose Type (DP) ^2^	Reference
N_1222_Fo	- ^1^	155 °C		MCC (330)	[168]
N_1222_Fo/Formic Acid/H_2_O (92/2/6 wt%)	10	80 °C, 15 min		MCC (330)	[168]
N_1222_Fo/Formic Acid/H_2_O (92/2/6 wt%)	4	80 °C, 15 min		Borregaard LVU pulp (1370)	[169]
N_1444_Fo	- ^1^	80 °C		MCC (330)	[168]
N_222 Me(OEt)2_AcO	10	110 °C		MCC (225)	[170]
N_222 Me(OEt)3_AcO	10	110 °C		MCC (225)	[170]
N_1122_AcO	2	100 °C, 30 min		α-cellulose	[118]
N_11[Et(OEt)]2_AcO	18	80 °C		MCC (240)	[171]
N_11 Et(OEt) Me(OEt)_AcO	17	80 °C		MCC (240)	[171]
N_11[Me(OEt)]2_AcO	15	80 °C		MCC (240)	[171]
N_112 M(OEt)_AcO	13	80 °C		MCC (240)	[171]
TBAAcO/DMSO (15/85 by weight)	8	RT, 2 min		MCC (250)	[85]
TBAAcO/DMSO (15/85 by weight)	1	RT, 13 min		Cotton (4080)	[85]
TBAAcO/DMSO (33/67 by weight)	15	40 °C		MCC (330)	[83]
TBAAcO/DMSO (22/78 by weight)	10	40 °C		MCC (330)	[83]
TBAAcO/DMSO (11/89 by weight)	6	40 °C		MCC (330)	[83]
TBAAcO/DMSO/18-crown-6 (2/7/1 by weight)	12	40 °C		Pulp (830)	[120]
TBAAcO/DMSO/18-crown-6 (2/7/1 by weight)	20	40 °C		MCC (200)	[120]
TBAPrO/methylimidazole (25/75 by weight)	15.7	RT		MCC (229)	[180]
N_2226_AcO	22	90 °C		MCC (330)	[73]
N_2226_AcO/DMSO (80/20 by weight)	20	80 °C		MCC (330)	[73]
*N*,*N*-allylmethylmorpho- liniumAcO	17	80 °C, 20 min		MCC (789)	[181]
*N*,*N*-allylmethylmorpho- liniumAcO	28	100 °C		MCC (789)	[181]
*N*,*N*-allylmethylmorpho- liniumAcO	11	80 °C, 20 min		Metsä Serla (2082)	[181]
N_122 Me(OEt)_ alaninate	12	100 °C, 10 min		MCC	[182]
N_122 Me(OEt)_ alaninate	6	60 °C, 10 min		MCC	[182]
N_122 Me(OEt)_AcO	7	60 °C		MCC	[182]
N_122 Me(OEt)_Cl	10	120 °C, 48 h		MCC	[182]
N_122 Me(OEt)_/DMSO (25/75 by mol)	11	RT, 10 min		MCC	[182]
N_122 Me(OEt)_/DMSO (25/75 by mol)	22	RT, 6 h		MCC	[182]
di-ChCl malinate	<1	90 °C	MCC		[184]
N_1333_ levulinate	10	90 °C, 2 h	MCC (200)		[186]
N_1333_ levulinate/γ-valero- lactone (80/20 by weight)	20	90–110 °C	MCC (200)		[186]
N_1144_ itaconate	10	90–110 °C	MCC (200)		[186]
N_1144_ AcO	9.5	90–110 °C	MCC (200)		[186]

^1^ - No data given, ^2^ DP - average degree of polymerization (in brackets), where available

**Table 3:** Zero shear viscosities (*η_0_* ) of cellulose solutions (2.4 wt %), 20 °C [193].

2.4 wt % Cellulose Dissolved in	*η_0_*[mPa·s]
BuMeImCl	197,000
EtMeImAcO	2747
DMAc/BuMeImCl	1346
DMSO/N_2228_Cl	237
DMAc/LiCl	147
DMAc/N_2228_Cl	87
Acetone/N_2228_Cl	47

**Table 4:** Summary of the solubility of cellulose in quaternary ammonium halides.

Cellulose Solvent	Dissolved Cellulose [wt%]	Dissolution Conditions	Cellulose Type (DP) ^2^	Reference
N_11-14-Bz_ Cl	5	155 °C	MCC (330)	[187]
N_2226_ Cl	sw ^1^	120 °C	MCC (330)	[61]
N_2227_ Cl	10	120 °C	MCC (330)	[192]
N_2228_ Cl	15	120 °C	MCC (330)	[192]
N_2228_ Cl	15	120 °C	PHK pulps (288, 534)	[194]
N_2228_ Cl/DMSO (1/1 by weight)	10	60 °C, 1 h	MCC (330)	[192]
N_2228_ Cl/DMSO (1/1 by weight)	7.5	120 °C, 1 h	PHK pulp (534)	[194]
N_2228_ Cl/DMAc (1/1 by weight)	10	60 °C, 1 h	MCC (330)	[192]
N_2228_ Cl/DMAc (1/4 by weight)	5	60 °C, 1 h	MCC (330)	[192]
N_2228_ Cl/acetone	10	60 °C, 2 h	MCC (330)	[193]
N_2222_Cl/NaOH/acetamide/H_2_O (6/8/10/76 by weight)	8.7	−5 °C, 15 min	Wood fiber (648)	[197]
DMSO/TBAF·3H_2_O (85/15 by weight)	2.5	RT, 15 min	MCC (330), pulp (650), linters (950)	[173]
DMSO/TAAF·H_2_O (93/7 by weight)	1.5	RT, 15 min	MCC (175) cotton (920)	[109]
DMSO/Bz_2_Me_2_AF∙0.1H_2_O (93/7 by weight)	1.5	60 °C, 15 min	MCC (175) cotton (920)	[110]

^1^ sw - swelling, ^2^ DP - average degree of polymerization (in brackets), where available.

**Table 5:** Summary of the solubility of cellulose in QAE and DES.

Cellulose Solvent	Dissolved Cellulose [wt%]	Dissolution Conditions	Cellulose Type (DP) ^1^	Reference
TBMA DMPh	12.5	60–100 °C, 1 h	MCC (335)	[222]
TMEA DMPh	6.5	60–100 °C, 1 h	MCC (335)	[222]
TPMA DMPh	2.1	60–100 °C, 1 h	MCC (335)	[222]
N_222Al_Cl/oxalic acid (1/1 by mol)	6.5	110 °C, 2 h	Cotton linters (575)	[228]
ChCl/oxalic acid (1/1 by mol)	1	110 °C, 2 h	Cotton linters (575)	[228]
ChCl/imidazole (3/7 by mol)	2.5	110 °C, 1.5 h	Cotton linters (575)	[229]
ChCl/urea (1/2 by mol)	1.5	110 °C, 2 h	Cotton linters (575)	[229]
ChCl/lactic acid (1/10 by mol)	3	60 °C, 20 min	Fibrous cellulose	[230]

^1^ - DP - average degree of polymerization (in brackets), where available.

## Supplementary Materials

Supplementary File 1

## Abbreviations and Acronyms

AcO	Acetate
AGU	Anhydroglucose unit
Al	Allyl group
AlMeImCl	1-Allyl-3-methylimidazolium chloride
BuMeImCl	1-Butyl-3-methylimidazolium chloride
Bz	Benzyl group
CDI	*N*,*N*′-Carbonyldiimidazole
Cel	Cellulose
ChCl	Choline chloride
CMC	Carboxymethyl cellulose
DBN	1,5-Diazabicyclo[[4.3.0]non-5-ene
DBU	1,8-Diazabicyclo[[5.4.0]undec-7-ene
DFT	Density functional theory
DMAc	*N,N*-Dimethylacetamide
DMF	*N*,*N*-Dimethylformamide
DMPh	Dimethylphosphate
DMSO	Dimethyl sulfoxide
DP	Average degree of polymerization
DS	Average degree of substitution
DSC	Differential scanning calorimetry
EtMeImAcO	1-Ethyl-3-methylimidazolium acetate
Fo	Formate group
IL	Ionic liquid
MCC	Microcrystalline cellulose
MD	Molecular dynamics simulations
Me(OEt)	Methoxyethyl
MS	Molecular solvent
NMMO	*N*-Methylmorpholine *N*-oxide
NMP	*N*-Methyl-2-pyrrolidone
N_RRRR_	Quaternary ammonium ion, R = 1 (methyl), 2 (ethyl), Al (allyl), Bz (benzyl), Me(OEt) (2-methoxyethyl), e.g., N_222 Me(OEt)2_ = bis(2-methoxyethyl)triethyl ammonium
PHK	Prehydrolysis kraft
PrO	Proprionate group
QACl	Quaternary ammonium chloride
QAF	Quaternary ammonium fluoride
SA	Solvent acidity as determined by solvatochromic probes (also designated as α)
SB	Solvent basicity as determined by solvatochromic probes (also designated as β)
SEC	Size exclusion chromatography
SEM	Scanning electron microscopy
TBA	Tetra-(*n*-butyl)ammonium
TBAAcO	Tetra-(*n*-butyl)ammonium acetate
TBACN	Tetra-(*n*-butyl)ammonium cyanide
TBAF	Tetra-(*n*-butyl)ammonium fluoride
TBAOH	Tetra-(*n*-butyl)ammonium hydroxide
TBPOH	Tetra-(*n*-butyl)phosphonium hydroxide
TMG	Tetramethylguanidine, Tetramethylguanidinium
χ	Mole fraction
W	Mass fraction
ῦ	Wavenumber
