# Supplementary material for: Recent Advances in Solvents for the Dissolution, Shaping and Derivatization of Cellulose: Quaternary Ammonium Electrolytes and their Solutions in Water and Molecular Solvents
Source: Molecules. 2018 Feb 25;23(3):511. doi: 10.3390/molecules23030511 (PMC6017797; doi:10.3390/molecules23030511)
Supplement: Supplementary File 1 [file molecules-23-00511-s001.pdf]

molecules

Review

Supplementary Material to

Recent Advances in Solvents for Dissolution, Shaping and Derivatization of Cellulose: Quaternary Ammonium Electrolytes and their Solutions in Water and Molecular Solvents

Marc Kostag, [1] Kerstin Jedvert, [2] Christian Achtel, [3] Thomas Heinze, [3] Omar A. El Seoud [1,*]

[1] Institute of Chemistry, University of São Paulo, Av. Prof. Lineu Prestes 748, 05508-000 São Paulo, SP, Brazil; e-mail: marc_kostag@usp.br

[2] Bio-based Fibres, Swerea IVF, P.O. Box 104, SE-431 22 Mölndal, Sweden; e-mail: kerstin.jedvert@swerea.se

[3] Centre of Excellence for Polysaccharide Research, Institute of Organic Chemistry and Macromolecular Chemistry, Friedrich Schiller University of Jena, Humboldtstraße 10, 07743 Jena, Germany; e-mail: christian.achtel@uni-jena.de and thomas.heinze@uni-jena.de

* Correspondence: elseoud.usp@gmail.com; Tel.: +55-11-3091-3874

Definitions

tex

The mechanical properties of filaments or fibers are often given in the unit of cN/tex (centi-Newton per tex). Tex is a unit of linear mass density of fibers, threads and yarns and is defined as the mass in grams per 1000 meters [1] (pp. 132–137). It is also common to use the unit decitex (dtex), the mass in grams per 10000 meters. Knowing the density of the fibers, it is possible to convert dtex-values to fiber diameters (for circular cross-sections) in SI-units, and thus convert cN/tex to tenacity values in MPa.

Spinneret (or spinnerette, also sometimes called die)

A spinneret is a small, thimble-shaped, metal plate with fine holes which is used for spinning of man-made fibers. The spinning solution is forced through the holes to form fine filaments, which are then solidified by coagulation, evaporation or cooling. The properties of the spinneret, e.g., amount of holes, shape and diameter of holes etc. will determine the properties of the filaments produced (e.g., amount, diameter, cross-sectional shape).

Draw ratio

During fiber spinning, the filaments or fibers are elongated by passing through a series of pair of rollers, in order to improve the orientation and increase the strength. The process is called drawing as each pair of rollers is moving faster than the previous one [1] (pp. 132–137). The draw ratio is defined as the ratio of speed of the front roller to the back roller. A high draw ratio is desirable to produce thinner fibers with high mechanical strength.

Spinning stability

There is no general definition for spinning stability. However, for the spinning to be considered stable there should be no filament breaks during the process. It is also important that there are no large variations in thickness and shape of the filaments [2,3].

Ring spinning

Ring spinning is a continuous process of spinning fibers (e.g. cotton, wool, staple fibers) to make a yarn. The basic principle of producing a yarn has not changed much since the beginning of spinning over a thousand years ago, with organizing, parallelizing, drawing, and twisting of the fibers. Three subsequent process steps constitute ring spinning; slubbing, ring spinning (using a ring-shaped frame) and winding it onto bobbins [1] (pp. 95–120).

Polydispersity index

Dispersity can be defined as a measure of the heterogeneity of sizes of molecules or particles in a mixture. For polymers, such as polysaccharides, this is often shown by measurements of the molecular mass distribution. The polydispersity index is the ratio between the weight average to the number average molecular weight: M_w/M_n is defined as:

$$M_n = (\sum N_i M_i)/\sum N_i$$

where N_i is the number of molecules with molecular weight M_i.

And M_w is defined as:

$$M_w = (\sum w_i M_i)/\sum w_i = (\sum N_i M_i^2)/(\sum N_i M_i)$$

where the weight, w, is the weighting factor for each molecular weight species [4].

References:

1. Gries, T.; Veit, D.; Wulfhorst, B. *Textile Technology: An Introduction*; Hanser Pub Incorporated, **2015**; ISBN 978-1-56990-565-4.
2. Wirth, B.; Warnecke, M.; Seide, G. H.; Schmenk, B.; Gries, T. *Filament breaches during air-gap spinning*; International Business Press Publ., **2011**; pp. 38–39.
3. Demay, Y.; Agassant, J.-F. An Overview of Molten Polymer Drawing Instabilities. *Int. Polym. Process.* **2014**, *29*, 128–139, doi:10.3139/217.2832.
4. Holmberg, K.; Jönsson, B.; Kronberg, B.; Lindman, B. Polymers in Solution. In *Surfactants and Polymers in Aqueous Solution*; John Wiley & Sons, Ltd, **2002**; pp. 193–214, ISBN 978-0-470-85642-0.
